# Arabidopsis MATE45 antagonizes local abscisic acid signaling to mediate development and abiotic stress responses

**DOI:** 10.1002/pld3.87

**Published:** 2018-10-12

**Authors:** Nik Kovinich, Yiqun Wang, Janet Adegboye, Alexandra A. Chanoca, Marisa S. Otegui, Paige Durkin, Erich Grotewold

**Affiliations:** ^1^ Center for Applied Plant Sciences and Department of Molecular Genetics The Ohio State University Columbus Ohio; ^2^ Davis College of Agriculture, Natural Resources and Design West Virginia University Morgantown West Virginia; ^3^ Department of Botany and Department of Genetics University of Wisconsin‐Madison Madison Wisconsin; ^4^ Laboratory of Molecular and Cellular Biology University of Wisconsin‐Madison Madison Wisconsin; ^5^ Present address: Davis College of Agriculture, Natural Resources and Design West Virginia University Morgantown West Virginia; ^6^ Present address: Department of Molecular and Cellular Biology Harvard University Cambridge Massachusetts; ^7^ Present address: Cleveland Clinic Lerner College of Medicine Cleveland Ohio; ^8^ Present address: VIB‐UGENT Center for Plant Systems Biology Zwijnaarde Belgium; ^9^ Present address: West Virginia University School of Dentistry Morgantown West Virginia; ^10^ Present address: Department of Biochemistry and Molecular Biology Michigan State University East Lansing Michigan

**Keywords:** abscisic acid, anthocyanin, membrane transport, metabolic stress, signaling

## Abstract

Anthocyanins provide ideal visual markers for the identification of mutations that disrupt molecular responses to abiotic stress. We screened *Arabidopsis* mutants of ABC (ATP‐Binding Cassette) and MATE (Multidrug And Toxic compound Extrusion) transporter genes under nutritional stress and identified four genes (*ABCG25*,*ABCG9*,*ABCG5,* and *MATE45*) required for normal anthocyanin pigmentation. *ABCG25* was previously demonstrated to encode a vascular‐localized cellular exporter of abscisic acid (ABA). Our results show that *MATE45* encodes an aerial meristem‐ and a vascular‐localized transporter associated with the *trans*‐Golgi, and that it plays an important role in controlling the levels and distribution of ABA in growing aerial meristems and non‐meristematic tissues. MATE45 promoter‐GUS reporter fusions revealed the activity localized to the leaf and influorescence meristems and the vasculature. Loss‐of‐function *mate45* mutants exhibited accelerated rates of aerial organ initiation suggesting at least partial functional conservation with the maize ortholog *bige1*. The *aba2‐1* mutant, which is deficient in ABA biosynthesis, exhibited a number of phenotypes that were rescued in the *mate45‐1 aba2‐1* double mutant. *mate45* exhibited enhanced the seed dormancy, and germination was hypersensitive to ABA. Enhanced frequency of leaf primordia growth in *mate45* seedlings grown in nutrient imbalance stress was ABA‐dependent. The ABA signaling reporter construct *pRD29B::GUS* revealed elevated levels of ABA signaling in the true leaf primordia of *mate45* seedlings grown under nutritional stress, and gradually reduced signaling in surrounding cotyledon and hypocotyl tissues concomitant with reduced expressions of *ABCG25*. Our results suggest a role of MATE45 in reducing meristematic ABA and in maintaining ABA distribution in adjacent non‐meristematic tissues.

1

**SIGNIFICANCE STATEMENT**

The metabolite transporter MATE45 has a role in reducing local ABA levels in growing leaf meristems and non‐cell autonomously in maintaining ABA distribution in adjacent non‐meristematic tissues. The study provides insight into the role of ABA in apical meristem patterning, growth, and non‐cell autonomously in mediating the anthocyanin response to abiotic stress.

## 2 INTRODUCTION

Based on integrated models of economy, climate, and crop yield, climate change will cause a 17% reduction in the mean global crop yield by 2050 (Nelson et al., 2014). To avoid a shortage of food, feed, and natural products, agricultural biotechnology is needed to improve plant tolerance to abiotic stresses so that yields can continue to increase (Southgate, 2009). Recent studies have demonstrated the critical roles for transporters, particularly those involved in the mobilization of a variety of metabolites, in mediating plant responses to stresses (Kuromori et al., 2010; Serrano et al., 2013; Tian et al., 2015). Among a number of other functions, metabolite transporters mediate the inter‐ and intra‐cellular transport of small molecules to control stress signaling and metabolite homeostasis (Barbez et al., 2012; Burla et al., 2013; Xu, Kim, & Hwang, 2013). Yet, only a few studies have so far identified metabolite transporters involved in these processes (Bonnemain, Chollet, & Rocher, 2013; Boursiac et al., 2013; Hamamoto et al., 2015). ABCG25 is an ATP‐binding cassette (ABC) transporter that is required to stimulate the guard cell responses to dehydration via the transport of the hormone abscisic acid (ABA; Kuromori et al., 2010). ABA is a major hormone that induces metabolic and physiological responses to abiotic stresses (Cutler, Rodriguez, Finkelstein, & Abrams, 2010; Finkelstein, 2013; Yoshida, Mogami, & Yamaguchi‐Shinozaki, 2015). ABCG25 and the MATE‐type transporter DTX50 localize to the plasma membrane of vascular cells, the predominant cellular site of ABA biosynthesis (Endo et al., 2008), and at least in heterologous systems, independently catalyze the cellular export of ABA (Kuromori, Sugimoto, & Shinozaki, 2014; Kuromori et al., 2010; Zhang et al., 2014). During dehydration stress, ABCG25 and DTX50 export ABA from vascular cells to the apoplastic space, where ABA is taken‐up by extravascular cells, such as guard cells. ABA is imported into guard cells by ABCG40 and induces a change in guard cell shape that results in the closure of the stomatal pore (Kang et al., 2010), even though ABA can be synthesized in the guard cell (Bauer et al., 2013). ABA‐IMPORTING TRANSPORTER (AIT) 1 is a vascular‐localized plasma membrane protein that is required to mediate the guard cell response to dehydration in inflorescence stems (Kanno et al., 2012).

Abscisic acid also induces anthocyanin pigment biosynthesis (Gagné, Cluzet, Mérillon, & Gény, 2011; Loreti et al., 2008; Shen et al., 2014). Anthocyanin pigmentation in the vegetative tissues of plants is a hallmark response to abiotic stresses (Chalker‐Scott, 1999; Gould, 2004; Kovinich et al., 2014; Winkel‐Shirley, 2002). Anthocyanins enhance tolerance to dehydration and oxidative stress, possibly by acting as free radical scavengers (Nakabayashi, Mori, & Saito, 2014; Nakabayashi, Yonekura‐Sakakibara, et al., 2014; Williams, Spencer, & Rice‐Evans, 2004). Anthocyanins are synthesized on the cytoplasmic surface of the endoplasmic reticulum (ER; Winkel‐Shirley, 1999). The mechanisms by which anthocyanins and other flavonoids are taken‐up into the vacuole, their site of storage, include tonoplast‐spanning transporters (MATE or ABC) and/or cytoplasmic bodies that are taken‐up by the vacuole in some plant species (Francisco et al., 2013; Gomez et al., 2009; Marinova et al., 2007; Zhao et al., 2011), and by a mechanism that resembles microautophagy in the model plant *Arabidopsis thaliana* (Arabidopsis) (Chanoca et al., 2015). While MATE and ABC transporters have been implicated in the movement of anthocyanins across the tonoplast in maize and grapevine (Gomez et al., 2009; Goodman, Casati, & Walbot, 2004), the involvement of transporters in the vacuolar sequestration of Arabidopsis anthocyanins remains to be determined.

Nutrient imbalances often result in plant growth inhibition and in the induction of anthocyanins (Hsieh, Lam, van de Loo, & Coruzzi, 1998; Jiang, Gao, Liao, Harberd, & Fu, 2007; Kovinich, Kayanja, Chanoca, Otegui, & Grotewold, 2015; Solfanelli, Poggi, Loreti, Alpi, & Perata, 2006). For example, anthocyanin biosynthesis is rapidly induced in Arabidopsis seedlings when grown in water (or nitrogen deficient media) and 3% sucrose, which we named anthocyanin induction condition (AIC; Kovinich et al., 2014, 2015; Pourcel et al., 2010; Poustka et al., 2007). In this study, we exploited the anthocyanin pigmentation induced by AIC to screen for transporter mutant lines affected in the anthocyanin response to abiotic stress. We identified MATE45 as a transporter required for the ABA‐dependent induction of stress responses and developmental processes. Our results demonstrate that MATE45 reduces ABA signaling in growing leaf and flower meristems and maintains ABA levels in adjacent tissues, and suggest that these are critical for plant growth and response to abiotic stress. *MATE45* is the ortholog of maize *BIGE1* (Suzuki, Sato, Wu, Kang, & McCarty, 2015), linking lateral organ initiation and size regulation with abiotic stress responses. Based on the results presented, we propose that MATE45 is involved in a pathway that cellautonomously antagonizes local ABA signaling in meristematic and vascular tissues at least in part resulting in the cellular efflux of ABA to adjacent tissues such as epidermal cells.

## 3 EXPERIMENTAL PROCEDURES

### 3.1 Accessions

Sequences for the following cDNAs are available at GenBank: *MATE45* ^*long*^ (KT070848), *MATE45* ^*med*^ (KT070849), *MATE45* ^*short*^ (KT070850), and *mate45‐1* ^*long*^ (KT150057).

### 3.2 Plant materials and growth conditions

Arabidopsis T‐DNA mutant lines (see Supporting Information Table S1) were obtained from the Arabidopsis Biological Resource Center (ABRC, Columbus, OH, USA). All were in the Columbia‐0 ecotype. Immediately before plating, seeds were surface‐sterilized with 70% ethanol with 0.2% Triton X, rinsed three times with ethanol, and dried. For screening in AIC, 12 seeds per line were plated in 1 ml of AIC medium consisting of water containing 3% sucrose (w/v) in a 24‐well microtiter plate. After stratification at 4°C for 3 days, microtiter plates were incubated on a rotary shaker at 110 rpm under cool white fluorescent light (85–100 μmol m^−2^ s^−1^) at 22°C for 5 days prior to visualization using a Nikon SMZ‐1500 stereo microscope. Lines were selected if all seedlings exhibited a visible alteration in pigmentation compared to the wild‐type. For all other AIC experiments, ~100 seeds were grown in 35 × 10 mm Petri dishes containing 3.5 ml of AIC medium as indicated above. For chemical treatments, hormones from 1,000 times‐concentrated stocks dissolved in DMSO were injected into AIC solution 96 hr after transferring to light. Seedlings were scored for the visible emergence of true leaf primordia after 12 days light, when all growth had arrested. For true leaf primordia counting and anthocyanin measurement of seedling grown in AIC, and the germination timing assays, all seeds used were harvested at the same time from parent plants grown under identical conditions on Sunshine LC1 potting mix under long‐day conditions (16 hr light, 8 hr dark photoperiod) at 22°C. Seeds were used 1–1.5 months after harvest to normalize for maturation and desiccation effects on seed ABA levels. For GUS staining of germinated seeds, seeds were placed in water and transferred directly to light with no prior dark treatment at 4°C. For GUS staining of seedlings, seeds were imbibed at 4°C and grown in AIC as indicated above.

### 3.3 Cloning and vector construction

The MATE45^long^, MATE45^med^, and MATE45^short^ open reading frames (ORFs) were cloned from cDNA constructed from WT Arabidopsis seedlings grown in AIC for 4 days using directional gene‐specific primers that enabled amplicons to be BP recombined into the pDONR221 vector (Invitrogen). Total RNA was isolated using the Spectrum Plant Total RNA Kit (Sigma‐Aldrich). cDNA was synthesized from 0.5 μg of total RNA using the SuperScript II First‐Strand Synthesis enzyme (Invitrogen) according to the manufacturer's instructions. For primer pairs used in this study, see Supporting Information Table S2. For ectopic overexpression of *MATE45* in the *mate45‐1* mutant, the MATE45^long^‐pDONR221 construct was LR recombined with the expression vector pGWB5, containing 35S constitutive promoter and C‐terminal GFP tag (Nakagawa et al., 2007), and was transformed into *mate45‐1*. The transgenic line *35S:MATE45* ^*long*^ *‐GFP mate45‐1* was selected in the T2 generation by GFP fluorescence in the root. All constructs were sequenced to ensure that they were error‐free. For complementation analysis of *mate45‐1*, a 2.2 kb segment of the *MATE45* promoter (*pMATE45*), a 2.4 kb genomic segment encompassing the *MATE45* coding sequence, and a 1.4 kb segment of the putative 3′UTR were BP recombined into the vectors pDONR P4‐P1R, pDONR221, pDONR P2R‐P3 (Invitrogen), respectively. The three constructs were recombined into the plant transformation vector pH7m34GW (http:gateway.psb.ugent.be; Karimi, Depicker, & Hilson, 2007) using the LR Clonase II Plus Enzyme (Life Technologies), and the construct was transferred into *mate45‐1*. The transgenic line *MATE45 mate45‐1* was selected in the T2 generation based on the inflorescence bud and rosette phenotypes. To silence *MATE45* gene expressions, a 122 nt segment of the first exon that is common to all splice forms of *MATE45* was BP cloned into pDONR221. The segment lacked consecutive 21 nt identity to other Arabidopsis genes. The segment was recombined into the pHELLSGATE12 plant transformation vector (CSIRO, Australia) (Helliwell & Waterhouse, 2003) and transferred into the WT. Three RNAi lines (*siMATE45‐4*,* ‐30*, and *‐31*) were selected in the T2 generation based on their pink anthocyanin phenotypes in AIC. To determine the expression pattern of *MATE45,* a 2.2 kb segment of the *MATE45* promoter was amplified by PCR and BP recombined into pDONR221. The resulting clone was LR recombined into the pGWB3 plant transformation vector that contains GUS downstream of the recombination site (Nakagawa et al., 2007). All constructs were transformed into Arabidopsis using the floral dip method (Clough & Bent, 1998). PCR‐based genotyping was conducted using the REDExtract‐N‐Amp Plant PCR Kit (Sigma‐Aldrich).

### 3.4 Germination assays

For germination assays, 50 sterilized seeds were plated on half‐strength MS, 0.5% agar medium containing 1% sucrose (w/v) and different concentrations of ABA. After stratification for 3 days at 4°C, germination was scored based on radicle protrusion. The mean and standard deviation were determined for three independent experiments. For dehydration assays and phenotyping experiments, seeds were transferred from plates lacking ABA to Sunshine LC1 potting mix, and grown for 2 months under long‐day conditions as indicated above.

### 3.5 Phenotypic analyses

Plants were grown on soil for 2 months as described above. For silique measurements, all siliques on the apical meristem were measured and the longest seven measurements were included in the comparison. Inflorescence buds from the apical meristem were stored in 70% ethanol until counting. The number of buds was determined at 7.5× total magnification. The fresh weight of rosettes was determined immediately after excision from the roots using an analytical scale. The inflorescence stems 1 cm above the soil were counted. Seven plants were used per experiment. Two independent experiments were performed.

### 3.6 Gene expression and localization of promoter activity

RNA was isolated from seedlings grown in AIC or on soil using the Spectrum Plant Total RNA Kit (Sigma‐Aldrich). For quantitative reverse transcriptase‐polymerase chain reaction (qRT‐PCR), RNA samples (500 ng) were treated with DNase I (Amplification grade; Invitrogen) to remove contaminating DNA. First‐strand cDNA was synthesized using Superscript II Reverse Transcriptase (Invitrogen). Parallel reactions were performed in the absence of Superscript II to test for genomic DNA contamination. Gene expressions from each cDNA sample were normalized to the endogenous reference *ACTIN2*. Reactions (10 μl) consisted of 0.4 μl of first‐strand cDNA (or untreated RNA), 250 nM of forward and reverse primers, and 5 μl of the iQ SYBR Green Supermix (BioRad). qRT‐PCR was performed on cDNA from four biological replicates or untreated RNA using a CFX96 thermal cycler (BioRad). To verify the specificity of the qRT‐PCR reactions, melting curves were determined subsequent to each reaction, and RT‐PCR products for each primer set were fractionated on agarose gels prior to qRT‐PCR.

To determine the localization of *pMATE45* activity, all GUS staining was performed for 24 hr using the whole‐mount protocol of Weigel & Glazebrook, 2002. Seedlings were imaged using an SMZ1500 stereomicroscope equipped with a Digital Sight DS‐Fi1 camera (Nikon).

### 3.7 Pigment and metabolite analyses

For pigment analysis, samples grown in AIC were harvested on ice, lyophilized for 3 days, and then extracted with 50% methanol 3% formic acid (v/v) (50 μg/μl) at room temperature overnight on a rotary shaker. Extracts were diluted with two volumes of 3% formic acid (v/v) in water prior to being passed through a 0.2 μM filter (Nanosep ODM02C35). Absorbance of the filtrate was analyzed at 532, 657, and 350 nm using a spectrophotometer (Nano Drop ND‐1000). For anthocyanin pigmentation, the formula Abs532–0.25·Abs657 was used to account for chlorophyll absorption at 532 nm (Mancinelli, 1990). Anthocyanin compositions in extracts were analyzed by HPLC‐PDA as described previously (Kovinich et al., 2014). Analysis of ABA was done using the method of Forcat, Bennett, Mansfield, & Grant, 2008. However, only ~5 mg of lyophilized tissue was extracted, and the final extract was resuspended in 100 μl of 10% MeOH (v/v) 1% Acetic Acid (v/v), and 20 μl was injected for MRM analysis by LC‐MS^n^. Metabolite separation was achieved using an Agilent 1290 Binary Pump and a Waters Symmetry C18 column (4.6 × 75 mm, 100 Å, 3.5 μm) held at 30°C. Mobile phases were 0.1% acetic acid in acetonitrile (A) and 0.1% acetic acid in water (B). The flow rate was 0.8 ml min^−1^. The gradient was held at 15% (A) for 1 min, then increased to 50% over 8 min, then to 80% over 3 min, at which point the gradient was returned to 15% and held for 3 min prior to the subsequent injection. MS^n^ was performed using a hybrid triple‐quadrupole/ion trap mass spectrometer QTRAP 5500 from AB Sciex. For MRM and MS^n^ settings, see Supporting Information [Link], [Link]. Data were processed using Analyst 1.6.1 software.

### 3.8 Transporter assays

The *Escherichia coli* mutant *acrB* deficient in drug efflux capability was obtained from the NBRP (National BioResource Project; National Institute of Genetics, Mishima, Japan). To test for metabolite transporter activity, the Arabidopsis MATE45 cDNAs and the control florescent protein Venus were recombined into the pDEST42 expression vector (Invitrogen), and the constructs were transformed into *acrB* cells. Transformants were selected on LB agar plates containing 100 μg ml^−1^ carbenicillin. Transformants were grown to saturation (16 hr) at 30°C in LB liquid medium containing carbenicillin and one mM IPTG (isopropyl‐β‐D‐thiogalactopyranoside). On ice, cultures were diluted to an absorbance at 600 nm of 0.400–0.410. Five microliter of culture was transferred to 1 ml LB containing 100 μg ml^−1^ carbenicillin and IPTG, with or without 1 mM Tetrabutylammonium chloride hydrate (TBA; Sigma) in a 24‐well multi‐titer plate. Cells were cultured at 30°C for 48 hr and the absorbance at 600 nm was measured at 20 min intervals using a Synergy HT Multi‐Mode Microplate Reader (BioTek).

### 3.9 Localization of GFP fusions by confocal microscopy

*Arabidopsis* seedlings were grown under AIC and imaged 5 days after germination. For colocalization of MATE45‐GFP with subcellular markers, images were taken in a Zeiss LSM 780 system. GFP was excited at 488 nm and emission collected at 493–536 nm, mCherry was excited at 561 nm and emission collected at 588–695 nm. Colocalization analysis was performed using the Coloc2 plugin for Fiji (Schindelin et al., 2012). For the brefeldin A (BFA) treatment, plants were incubated with 50 μM BFA for 2.5 hr and 4 μM FM4‐64 was added during the last 30 min of incubation. Images were taken in a Zeiss LSM 510 Meta microscope. GFP and FM4‐64 were excited at 488 nm; GFP emission was collected at 500–530 nm and FM4‐64 emission at 650–710 nm.

## 4 RESULTS

### 4.1 A high‐throughput visual screen identified four transporter gene mutants affected in the anthocyanin response to abiotic stress

**Figure 1:**
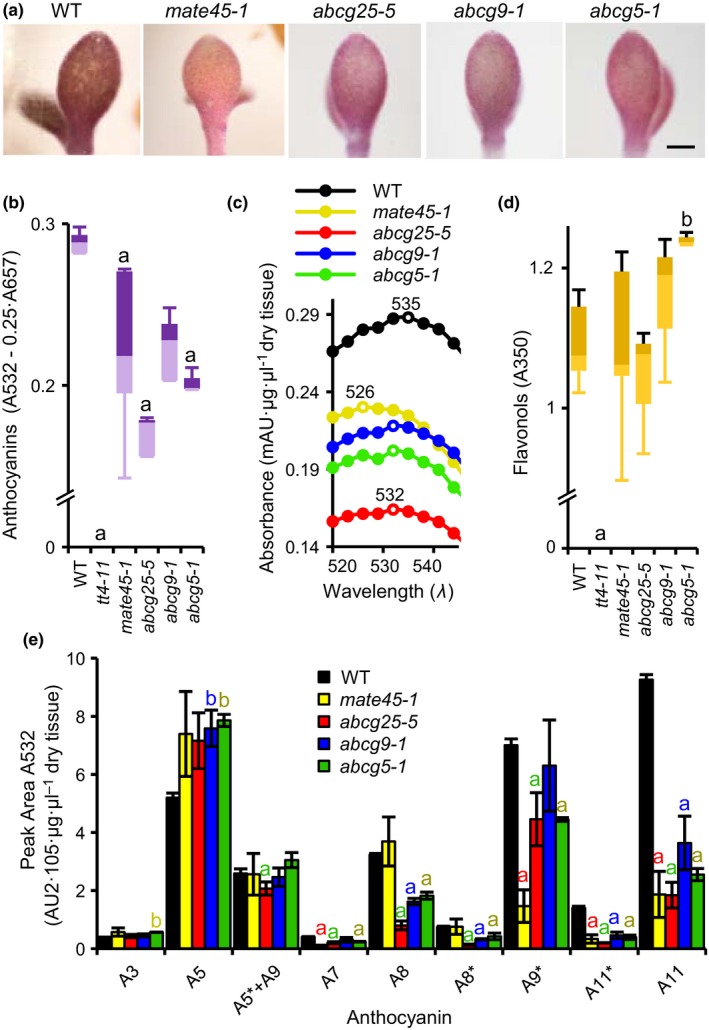
A high‐throughput screen identifies metabolite transporter genes involved in the anthocyanin response to anthocyanin induction condition (AIC) stress. (a) Pale anthocyanin pigmentation phenotypes of the four transporter mutants when grown under AIC condition. The mutants *mate45‐1* (CS865060), *abcg25‐5* (SALK_128873C), *abcg9‐1* (SALK_045397C), and *abcg5‐*1 (SALK_074250C) had pink cotyledon color, whereas the wild‐type (WT) had purple cotyledons. 24‐well multi‐titer plates were incubated on a rotary shaker under 24 hr light for 5 days. (b) Total anthocyanin absorbance of methanolic extracts. *tt4‐11* is a flavonoid biosynthesis mutant. Error bars represent the standard error of the mean (*n* = 3). ^a^Less than control, ^b^greater than control, *p *<* *0.05; two‐tailed Student's *t* test. (c) Spectral scan of methanolic extracts in the absorbance range of anthocyanins. Open circles and numbers above curves indicate absorbance maxima. (d) Total flavonol absorbance of methanolic extracts. (e) Relative anthocyanin amounts in methanol extracts determined by HPLC‐PDA

To search for metabolite transporters that affect anthocyanin pigmentation under abiotic stress either directly by affecting their vacuolar sequestration, or indirectly by modulating signaling molecules that participate in the stress response, we screened T‐DNA insertion mutants of 55 MATE and 114 ABC genes grown under AIC for a visible alteration in anthocyanin pigmentation (see Supporting Information Table S1). To better understand the genetics of the population, we genotyped 94 lines and found that ~57% were homozygous mutant (see Supporting Information Table S1). Four of the lines identified as homozygous mutants had pink cotyledons, in contrast to the normal purple color of the wild‐type (WT) (Figure 1a). According to the sequence of the genomic DNAs flanking the T‐DNAs, the T‐DNA elements were inserted in the third exon of *ABCG25* (At1g71960), the third exon of *ABCG9* (At4g27420), the 5′‐UTR of *ABCG5* (At2g13610), and the second exon of At1g71870 (see Supporting Information Figure S1). Based on the hierarchical position of the predicted protein encoded by At1g71870 on the MATE family phylogenetic tree (see Supporting Information Figure S2), we called this gene *MATE45*.

The four mutant lines (*mate45‐1*,* abcg25‐5*,* abcg9‐1,* and *abcg5‐1*) were grown to maturity, and the pigmentation phenotype in AIC was confirmed in the subsequent generation. To quantitatively evaluate the anthocyanin levels in the mutants, we measured the absorbance of methanol extracts by spectrophotometry, and compared them to that of the WT (Figure 1b). The four mutants had significantly lower levels of anthocyanins than WT (*p *<* *0.05; two‐tailed Student's *t* test; Figure 1b), and different anthocyanin absorbance maxima (Figure 1c). In contrast, the levels of flavonols, which generally cause a deepening of the anthocyanin color by functioning as copigments, were not significantly reduced (Figure 1d). As a control, similar analysis was performed with a chalcone synthase mutant that completely lacks anthocyanins, *tt4‐11*; as expected, no anthocyanins or flavonols were detected in this mutant (Figure 1b,d). High performance liquid chromatography‐photodiode array (HPLC‐PDA) of extracts evaluated at 532 nm showed that the four mutants contained reduced levels of A11 (Tohge et al., 2005), the most abundant and most highly decorated anthocyanin in WT seedlings (Figure 1e). Highly decorated cyanidin derivatives such as A11 and its isomer A11* contribute a deeper purple hue to tissues than less decorated cyanidin derivatives. Together, these results show that the pale pink color of the four mutants is a consequence of the selective reduction of specific anthocyanins, mainly A11.

### 4.2 Genetic and functional characterization of *MATE45*

Of the four transporter mutants identified by our screen, *mate45‐1* exhibited the most dramatic visible change in anthocyanin pigmentation compared to WT (Figure 1a). We amplified and sequenced the *mate45‐1* allele and confirmed the location of the predicted T‐DNA insertion site in the final exon (See Supporting Information Figure S3A). *MATE45* transcripts were not detected in *mate45‐1* by (qRT‐PCR using primers that targeted a region downstream of the T‐DNA insertion (See Supporting Information Figure S3B, primer sets 2 and 3). However, qRT‐PCR using primer pairs that targeted various regions upstream of the T‐DNA insertion revealed that *MATE45* transcripts were still highly expressed (See Supporting Information Figure S3B, primer sets 1, 4, 5, and 6). Sequencing of cDNA clones of *mate45‐1* identified a premature stop codon inside the T‐DNA that resulted in the deletion of 61 amino acids spanning the predicted C‐terminal transmembrane domain (TMD) to the cytosolic tail of the protein (see Supporting Information Figure S4). This region showed 76% amino acid similarity to the cation‐binding pocket of the *Vibrio cholerae* NorM MATE transporter, important for positioning catalytic residues required for transporter activity (He et al., 2010) (see Supporting Information Figure S3).

We similarly cloned and sequenced *MATE45* cDNAs from WT seedlings grown for 4 days in AIC, and identified three alternative splice forms: *MATE45* ^*long*^, *MATE45* ^*med*^, and *MATE45* ^*short*^ (See Supporting Information Figure S3A). The same splice forms were isolated from 4 day‐old seedlings grown on soil under optimal conditions (see METHODS), indicating that they were not specifically produced in response to stress. *MATE45* ^*long*^ and *MATE45* ^*med*^ were predicted to encode proteins with 12 TMDs helices (see Supporting Information Figure S3), similar to the structures determined by X‐ray crystallography for NorM and for the functional core of mammalian MATEs (He et al., 2010; Zhang et al., 2012). In contrast, *MATE45* ^*short*^ was predicted to encode a protein with eight TMDs.

To determine whether the reduced levels of anthocyanins in *mate45‐1* seedlings (Figure 1b) could be attributed to the defect in the *MATE45* gene, we characterized an additional homozygous T‐DNA insertion allele, *mate45‐2* (See Supporting Information Figure S3; see Supporting Information Table S1), and two RNA interference lines, *siMATE45‐30* and *siMATE45‐31. mate45‐2* harbored an insertion in the intron and had significantly reduced mRNA and anthocyanin levels (See Supporting Information Figure S3C). *siMATE45‐30* and *siMATE45‐31* each had an approximately 50% reduction in both *MATE45* mRNA levels (See Supporting Information Figure S3C) and anthocyanin content compared to WT (See Supporting Information Figure S3D). To test if MATE45 can rescue the observed defects in anthocyanin accumulation, we transformed *mate45‐1* with the genomic copy of WT *MATE45* including a region spanning 2.2 kb upstream of the translation start codon (*proMATE45*::*MATE45*). *mate45‐1* plants harboring *proMATE45*::*MATE45* accumulated significantly more *MATE45* transcripts than WT, and exhibited restored anthocyanin content to levels even greater than those present in WT (See Supporting Information Figure S3D). We generated an additional line that overexpressed *MATE45* from the constitutive 35S promoter (*pro35S*::*MATE45* ^*long*^ *‐GFP*). Interestingly, although *pro35S::MATE45* ^*long*^ *‐GFP mate45‐1* seedlings expressed *MATE45* at levels higher than WT (See Supporting Information Figure S3C), their anthocyanin levels were not significantly different from WT, suggesting that the spatial and/or temporal expression patterns conferred by *proMATE45* are required for increased anthocyanin accumulation.

**Figure 2:**
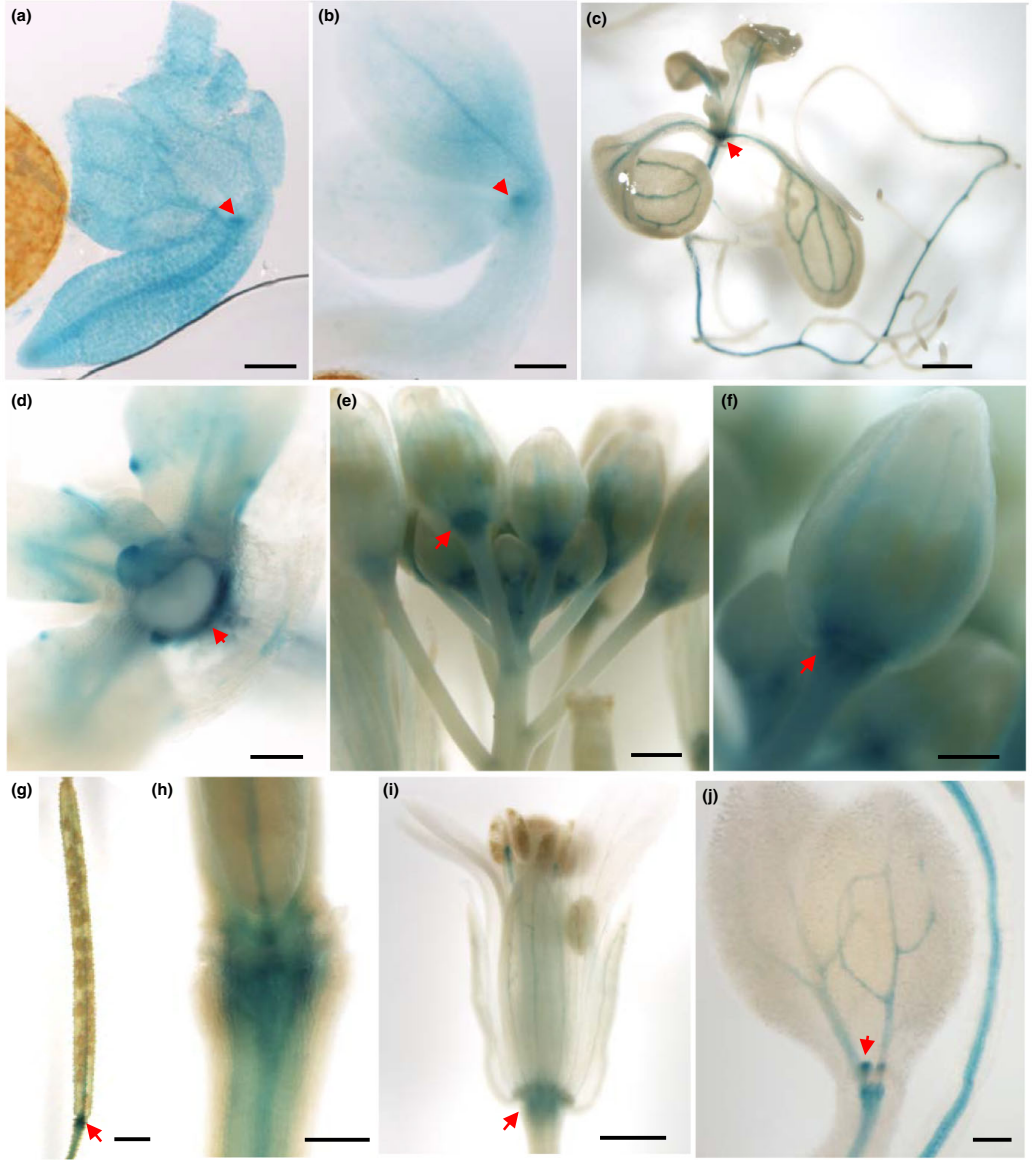
Tissue localization of *proMATE45* activity. (a) Seed imbibed for 24 hr in water. Seed coat was removed to allow visualization. Arrow indicates emerging true leaf primordium. Scale: 125 μm (b–e, seedlings/seeds were grown/imbibed in liquid 1/2MS medium 1% sucrose under 24 hr light at 22°C). (b) Seed imbibed for 48 hr. Seed coat was removed to allow visualization. Arrow indicates emerging true leaf primordium. Scale: 125 μm. (c) Two‐week‐old seedling. Arrow indicates region of emerging leaf primordia. Scale: 1 mm. (d) Enlargement of region of emerging leaf primordium. Arrow indicates base of emerging primordium. Scale: 125 μm. (e) Apical inflorescence meristem. Arrow indicates the flower bud‐pedicel junction. Scale: 500 μm. (f–j, plants were 2 months‐old grown on soil under 16 hr light, 8 hr dark at 22°C). (f) Enlargement of flower bud‐pedicel junction. Sclae: 250 μm. (g) Silique. Arrow indicates silique‐pedicel junction. Scale: 1 mm. (h) Enlargement of silique‐pedicel junction. Scale: 250 μm. (i) Opened flower. Arrow indicates flower‐pedicel junction. Scale: 500 μm. (j) Seedling grown for 6 days in anthocyanin induction condition. Arrow marks the emerging leaf primordia. Scale: 125 μm

To determine the spatiotemporal pattern of *proMATE45* activity, we introduced a *GUS* reporter driven by (*proMATE45::GUS*) into WT plants. Twenty‐four hours after seeds were transferred to light, GUS staining was observed in leaf primordia and vasculature (Figure 2a). By 48 hr after light, the staining in the root vasculature generally faded and staining was concentrated at the emerging leaf primordium (Figure 2b). After 2 weeks of development, staining was detected throughout the vasculature, but was most intense at the base of the rosette where new leaves were emerging (Figure 2c,d). In growing flower buds and siliques, staining pronounced at the base of the organs and in the vasculature (Figure 2e–h). As the flower developed, staining became less intense (Figure 2i). Together, these results show that, in the absence of stress treatments, *MATE45* is mostly expressed in growing meristems and in the vasculature. In seedlings grown in AIC, GUS staining was restricted to emerging leaf primordia and the vasculature, similar to seedlings grown in normal condition (Figure 2j). Since anthocyanins accumulate predominantly in the epidermal cells of WT seedlings grown in AIC (Kovinich et al., 2015) and *mate45* mutants exhibited a pale anthocyanin pigmentation phenotype, the localization of *proMATE45* activity to the vasculature suggested a non‐cell autonomous function of *MATE45*, rather than a direct role in anthocyanin transport in the epidermal cells.

### 4.3 Transporter activity of MATE45

To determine whether *MATE45* encoded transporter activity, we cloned ORFs of the three splice forms into the *pDEST42* vector under control of the *T7 lacO* inducible promoter, and tested their ability to confer tolerance to the toxin tetrabutylammonium (TBA) when expressed in the *E. coli* mutant *acrB* (Du et al., 2014; Ma et al., 1995). TBA is a synthetic toxin that is generally recognized as a substrate by MATE proteins, and its toxicity toward *E. coli* is enhanced by the *acrB* mutation that confers a defect to the *AcrAB–TolC* multidrug efflux system (Seo et al., 2012).

**Figure 3:**
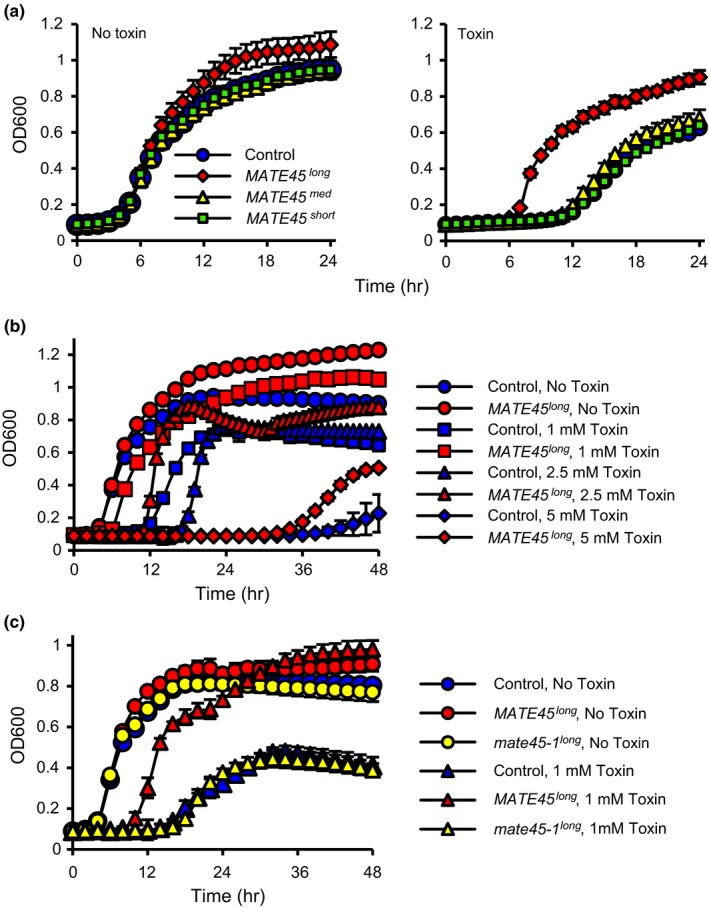
MATE45^long^ has metabolite efflux activity. (a) Mutant *Escherichia coli* strain *acrB* that is defective in the AcrAB‐TolC efflux system was transformed with ORFs *MATE45* ^*long*^, *MATE45* ^*med*^, *MATE45* ^*short*^ or yellow florescent protein (Control), and grown in the presence or absence of 1 mM of the toxin tetrabultylammonium (TBA). Bacterial growth absorbance is indicated on the vertical axis and time after initiating the culture is indicated on the horizontal axis. In the absence of toxin, all genotypes reached exponential growth at the same time, ensuring that equal numbers of bacteria were plated for each genotype. Only the *MATE45* ^*long*^ ORF provided tolerance to TBA toxin, as demonstrated by the failure to delay exponential growth. (b) *acrB* transformed with the *MATE45* ^*long*^ ORF or yellow florescent protein (Control) was grown in the presence or absence of different concentrations of the toxin TBA. *MATE45* ^*long*^ provided tolerance to the toxin, as demonstrated by earlier times until exponential phase growth in the present of the toxin. (c) mate45‐1^long^ lacked MATE efflux activity. *acrB* was transformed with the ORF *mate45‐1* ^*long*^, *MATE45* ^*long*^, or yellow florescent protein (Control) and grown in the presence or absence of 1 mM TBA toxin. *mate45‐1* ^*long*^ failed to provide tolerance to the TBA, as demonstrated by similar growth curve compared to the control

Tetrabutylammonium significantly reduced the growth of *acrB* cells expressing *MATE45* ^*short*^ and *MATE45* ^*med*^, whereas cells expressing *MATE45* ^*long*^ were significantly more resistant than the *acrB* control over a broad concentration range (Figure 3a,b). In contrast, the truncated CDS encoded by the *mate45‐1* mutant allele was unable to confer tolerance to TBA (Figure 3c). These results demonstrate that MATE45^long^ has metabolite transporter activity but the alternative splice forms *MATE45* ^*short*^ and *MATE45* ^*med*^ and the mutant allele *mate45‐1* lack transporter activity.

### 4.4 MATE45 localizes to the Golgi

**Figure 4:**
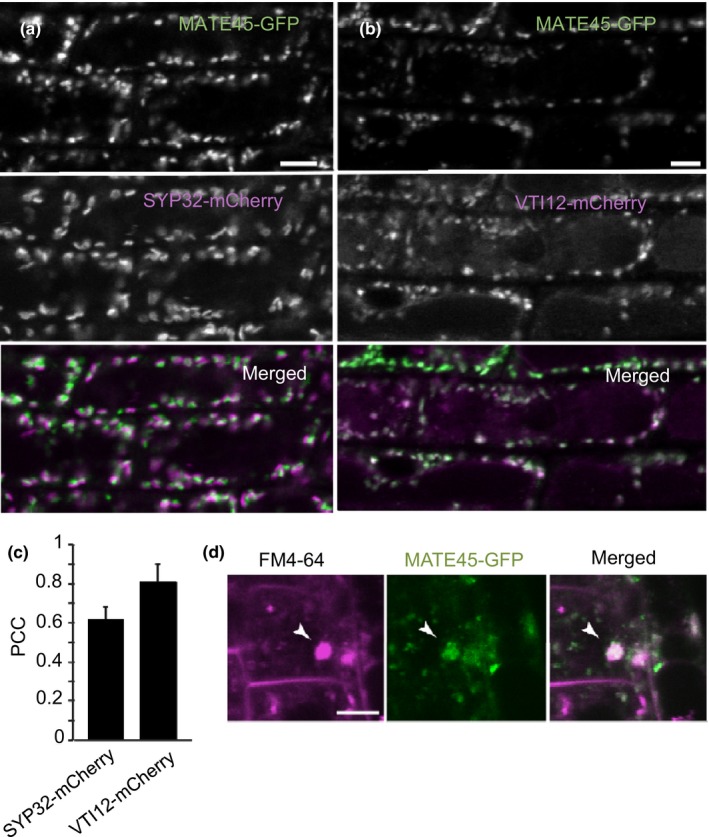
Subcellular localization of MATE45‐GFP in Arabidopsis root cells. (a) Coexpression of MATE45‐GFP with the Golgi marker SYP32‐mCherry. (b) Coexpression of MATE45‐GFP with the trans‐Golgi network marker VTI12‐mCherry. (c) Pearson's Correlation Coefficient (PCC) analysis of colocalization between MATE45‐GFP and SYP32‐mCherry and VTI12‐mCherry. (d) Upon brefeldin A (BFA) treatment, MATE45‐GFP is relocated to BFA compartments (arrowhead) together with the membrane dye FM4‐64. Scales: 5 μm

To determine the subcellular localization of MATE45, we used confocal microscopy to examine plants expressing MATE45 fused to a C‐terminal fluorescent tag. We were unable to detect a fluorescence signal in plants expressing *proMATE45::MATE45‐YFP*. Therefore, by confocal microscopy we analyzed the plants harboring *pro35S::MATE45* ^*long*^ *‐GFP*. We observed GFP‐positive cytoplasmic organelles of approximately 1 μm in diameter in root cells (Figure 4a,b). To determine the identity of these structures, we coexpressed p35S::*MATE45‐GFP* with several subcellular markers (Geldner et al., 2009). When MATE45‐GFP was coexpressed with the trans‐Golgi network (TGN) marker VTI12‐mCherry, both fluorescent signals colocalized (Pearson's correlation coefficient = 0.81), whereas we observed a lower degree of colocalization between MATE45‐GFP and the Golgi marker SYP32‐mCherry (Pearson's correlation coefficient = 0.62; Figure 4c). In most cases, the MATE45‐GFP signal was adjacent and partially overlapping with the SYP32‐mCherry‐labeled Golgi stacks, consistent with the localization of MATE45 to the TGN, and partially to the Golgi. To further confirm the localization of MATE45‐GFP, we treated *p35S:MATE45‐GFP* seedlings with BFA. BFA is a fungal toxin that blocks vesicle trafficking and induces the formation of membrane aggregates (BFA compartments) with TGN membranes in the center and Golgi membranes in the periphery (Grebe et al., 2003). Seedlings incubated for 2.5 hr in 50 μM BFA and 4 μM of the membrane dye FM4‐64 showed relocalization of most of the MATE45‐GFP signal to BFA compartment cores and some to the BFA compartment periphery (Figure 4d). While we cannot rule‐out that ectopically overexpressing MATE45‐GFP modified its protein localization slightly compared to the native protein, our results suggest that MATE45 localizes to the TGN and Golgi apparatus similar to its maize homolog BIGE1 (Suzuki et al., 2015).

### 4.5 MATE45 function in growth and development is ABA‐dependent

**Figure 5:**
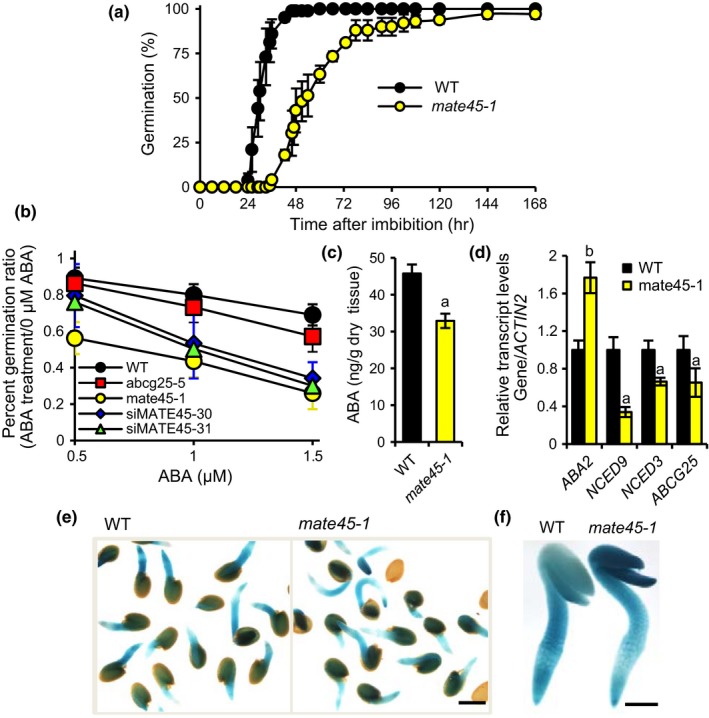
The growth and developmental phenotypes of MATE45 mutants are abscisic acid (ABA)‐dependent. (a) Germination of seeds on agar‐based media. Seeds were imbibed for 3 days at 4°C, germination rates following the imbibition treatment are shown. (b) Percent seed germination after 48 hr on agar media containing different concentrations of ABA. Germination was scored by the visible protrusion of the radical from the seed coat. The average from three plates of 50 seeds is shown. (c) Amount of ABA from seeds imbibed at 4°C for 24 hr measured by LC‐MS ^n^. Error bars represent the standard error of the mean (*n* = 4 biological replicates). ^a^Less than control, *p *<* *0.05; two‐tailed Student's *t* test. (d) qRT‐PCR measurements of gene expressions relative to *ACTIN2*. ^a^Less than control, ^b^greater than control, *p *<* *0.05; two‐tailed Student's *t* test. (e) Staining of ABA signaling marker *pRD29B:GUS* in germinating seeds. Cotyledons of mate45‐1 generally stained more intensely than cotyledons of the wild‐type. Scale 500 μm. (f) Seeds in (e) with seed coat removed. Scale 250 μm

During routine growth of plants for seed collection, *mate45‐1* exhibited enhanced seed dormancy or delayed germination in the absence of stress treatment. To investigate this effect further, we investigated rates of germination after imbibing seeds in the cold for 3 days at 4°C, which has been shown to remove endogenous ABA by promoting its catabolism to minimize ABA‐imposed dormancy (Millar et al., 2006). The seeds that were assayed were from *mate45‐1* and WT plants that were grown to maturity under identical conditions. Seeds were 1–1.5 months after harvest to normalize for maturation and desiccation effects on seed ABA levels. Three independent experiments using separate batches of seeds confirmed that *mate45‐1* showed delayed germination despite imbibition at 4°C (Figure 5a), suggesting a defect in germination.

Our screen identified *abcg25‐5*, corresponding to an ABA transporter mutant. *ABCG25* mutants were previously reported to exhibit increased sensitivity to ABA during germination (Kuromori et al., 2010). To determine whether *MATE45* mutants had increased ABA sensitivity, we tested germination rates in the presence of ABA treatment after 3 days imbibition at 4°C. The germination ratio of ABA‐treated to untreated seeds of each genotype yielded a similar slope over increasing ABA treatment concentrations (Figure 5b), indicating similar sensitivities to ABA. However, the mutants exhibited a lower germination ratio compared to the WT at each individual treatment concentration, suggesting that initial levels of ABA or ABA signaling were elevated. To measure ABA levels, *mate45‐1* and WT plants were grown for one generation under identical conditions, and ABA was quantified from seeds that were imbibed for 24 hr. Surprisingly, *mate45‐1* seeds had ~30% less ABA than WT (Figure 5c). This suggested that elevated localized levels of ABA or elevated signaling was causing feedback suppression of ABA biosynthesis. Consistent with this hypothesis, the expression levels of ABA biosynthesis genes *NCED9* and *NCED3* and the transporter *ABCG25* were significantly reduced in *mate45‐1* (Figure 5d). Introducing the ABA signaling marker *pRD29B::GUS* (Christmann, Hoffmann, Teplova, Grill, & Muller, 2005) into the *mate45‐1* mutant by crossing revealed more intense staining in the cotyledons relative to the root at 24 hr after germination, a staining pattern not observed in WT seedlings (Figure 5e,f). From these results, we conclude that the more sensitive phenotype of *mate45‐1* to ABA was due to enhanced localized ABA accumulation or signaling.

During development, in the absence of stress treatment, *mate45‐1* plants show reduced rosette biomass, increased numbers of inflorescence stems, and shorter siliques compared to the WT (Table 1, see Supporting Information Figure S5). To investigate to what extent these phenotypes are a consequence of *mate45‐1*, rather than resulting from indirect effects on anthocyanin (or possibly other flavonoid) accumulation, we generated *mate45‐1 tt4‐11* double mutant plants. Each of the growth phenotypes observed for *mate45‐1* was also observed in the double mutant (see Supporting Information Figure S5), demonstrating the developmental abnormalities were not a result of the altered anthocyanin or other flavonoid accumulations.

**Table 1:** Phenotypic comparison of *MATE45* mutants and transgenic lines to the WT

Line	*mate45‐1*	*MATE45 mate45‐1* a	*mate45‐2*	*siMATE45‐30*	*siMATE45‐31*	*mate45‐3*	*35S:MATE45* ^*long*^ *mate45‐1*	*abcg25‐5*	*aba2‐1*	*mate45‐1 aba2‐1*
MATE45 sequence	3′‐truncated	WT and 3′‐truncated	WT	ND	ND	ND	Long splice form and 3′‐truncated	ND	ND	3′‐truncated
MATE45 expression levels	Increased^b^	Increased	Reduced^c^	Reduced	Reduced	Increased (ectopic)	Increased (ectopic)	ND	ND	ND
Rosette fresh weight	Reduced	WT	Reduced	ND	ND	Reduced	Reduced	Reduced	Reduced	WT
Inflorescence stem number	Increased	WT	WT	Increased	Increased	WT	Increased	WT	Increased	WT
Apical flower bud number	Increased	Reduced	Increased	ND	ND	WT	Increased	WT	WT	WT
Silique length	Reduced	Increased	Reduced	ND	ND	WT	Reduced	WT	Reduced	Intermediate^b^

*Notes*

ND = not determined.

^a^Complemented line. ^b^Statistically greater than wild‐type (WT); two‐tailed Student's *t* test *p *<* *0.05. ^c^Statistically less than WT (WT); two‐tailed Student's *t* test *p* < 0.05.

To test for dependence of the growth phenotypes on ABA, we generated the *mate45‐1 aba2‐1* double mutant. In this double mutant, all the developmental and growth defects were fully rescued, except for silique length, which was only partially rescued (see Supporting Information Figure S5). This demonstrated that constitutively reducing the levels of ABA biosynthesis could offset the excessive localized ABA signaling conferred by *mate45‐1*. We therefore conclude that the growth and developmental phenotypes observed in *mate45‐1* are caused by mis‐signaling or mis‐localization of ABA and not anthocyanins or other flavonoids.

### 4.6 Ectopic ABA accumulation in *mate45* promotes accelerated rates of leaf primordia initiation

**Figure 6:**
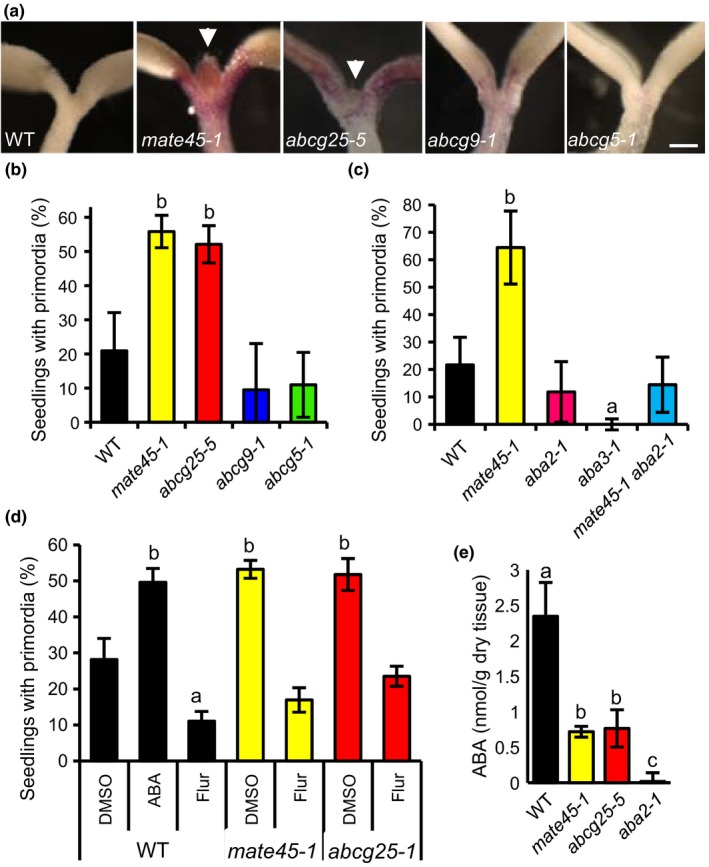
Accelerated true leaf primordia initiation of *mate45‐1* in anthocyanin induction condition (AIC) is abscisic acid (ABA)‐dependent. (a) True leaf primordia growth phenotype of lines identified by our screen. *mate45‐1* and *abcg25‐5* seedlings generally had visible true leaf primordia at 10 dag in AIC. Scale 250 μm. Percentage of seedlings that had visible true leaf primordia at 12 dag in AIC: (b) Mutants identified by our screen (*n* = 4 biological replicates). (c) ABA‐deficient mutants *aba2‐1* and *aba3‐1*, and the *mate45‐1 aba2‐1* double mutant. *mate45‐1 aba2‐1* restored wild‐type rates of primordia initiation (*n* = 4 biological replicates). (d) Seedlings treated with 10 μM ABA biosynthesis inhibitor fluridone (Flur), 3 μM ABA, or solvent (DMSO) (*n* = 4 biological replicates). (e) ABA amounts from lyophilized seedlings at 5 dag in AIC. Two‐way ANOVA, Tukey post hoc test (*p* < 0.01); different letters show significant differences (*n* = 4 biological replicates)

After growing seedlings for 10 days under the stress of AIC, most *mate45‐1* seedlings developed true leaf primordia prior to arresting growth, whereas WT seedlings predominantly arrested growth prior to the developing the primordia (Figure 6a,b). The relative frequencies of true leaf primordia among genotypes were highly reproducible when the seeds were assayed were from plants grown and harvested together under identical conditions. Thus, the leaf primordia assay in AIC represented a robust new approach to quantitatively analyze rates of leaf primordia initiation. To ensure that accelerated primordia development was caused by the *mate45‐1* allele, we determined that *proMATE45:*:*MATE45* restored WT frequencies of leaf primordia (see Supporting Information Figure S6A). Similar frequencies of primordia development were observed for *mate45‐1* and *mate45‐1 tt4‐11*, indicating that the phenotype was not dependent on anthocyanins/flavonoids (see Supporting Information Figure S6B).

Since most developmental phenotypes of *mate45‐1* were rescued by *aba2‐1*, we investigated whether primordia initiation rates in AIC were dependent on ABA. Consistent with an involvement of ABA in the establishment of the *mate45‐1* phenotypes, *mate45‐1 aba2‐1* had normal leaf primordium initiation rates (Figure 6c). Furthermore, *mate45‐1* treated with 10 μM fluridone (Flur), an ABA biosynthesis inhibitor, had rates indistinguishable from those of the WT (Figure 6d). In contrast, treatment of WT seedlings with 3 μM ABA increased primordia growth frequencies to levels equivalent to *mate45‐1* (Figure 6d). As with imbibing *mate45‐1* seeds under non‐stressed conditions (Figure 5c), *mate45‐1* seedlings had reduced levels of ABA compared to the WT in AIC (Figure 6e). These results suggest that mis‐localized ABA accumulation is responsible for the increased leaf primordia initiation rates of *mate45*.

**Figure 7:**
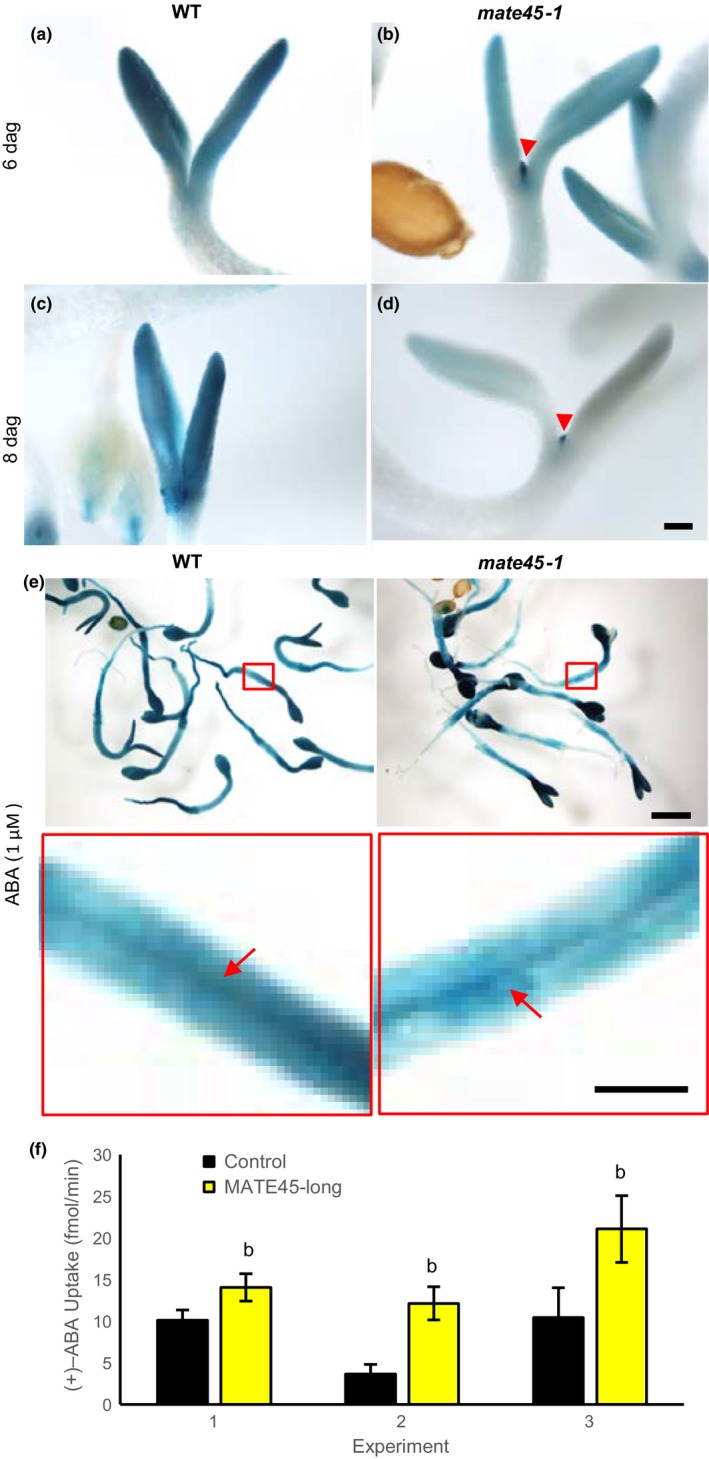
Ectopic localization of abscisic acid (ABA) signaling in *mate45‐1 pDR29B:GUS* and (+)‐ABA uptake. (a–d) Seedlings of wild‐type (a, c) and *mate45‐1* (b, d) expressing the ABA signaling marker *pDR29B:GUS* imaged six dag (a, b), and eight dag (c, d) in anthocyanin induction condition (AIC). Arrowheads mark regions of ectopic staining in *mate45‐1 pRD29B:GUS*. Scale: 125 μm. (e) Response to ABA treatment. Seedlings grown in AIC for 5 days were treated with 1 μM ABA for 24 hr by application to the medium. Upper panels scale: 1 mm. Lower panels are enlargements of the boxed areas shown upper panels. Arrows indicated vasculature. *mate45‐1* exhibited relatively weak staining in extravascular tissues of the hypocotyl and cotyledon. Scale: 250 μm. (f) Rate of (+)‐ABA uptake into acrB cells expressing MATE45long. Cells expressing MATE45long (yellow bars) or Venus fluorescent protein as a non‐transporter control (black bars) were pelleted and resuspended in medium containing 0.5 μM (+)‐ABA. After 0 or 30 min of uptake cells were re‐pelleted and extracted for analysis (+)‐ABA amounts by LC‐MS in MRM mode. Bars represent the rate of (+)‐ABA uptake calculated as the amount of (+)‐ABA at T = 30 min subtracted by the amount adhered to the cells at 0 min divided by the 30 min incubation time. bGreater than control, *p* < 0.05; two‐tailed Student's *t* test

To determine whether *mate45‐1* had altered localization of ABA signaling prior to leaf primordia initiation, we compared the staining patterns of *pRD29B::GUS* between *mate45‐1* and WT seedlings. At 4 days after germination (dag), the pattern of GUS activity was similar in *mate45‐1* and WT seedlings, with more intense staining observed in the cotyledons compared to the hypocotyls and roots (see Supporting Information Figure S7). However, by 5 dag, the differences between genotypes became apparent when staining in *mate45‐1* was more uniform in intensity between hypocotyl and root (see Supporting Information Figure S7). By 6 dag, ectopic staining was observed in emerging leaf primordia of ~25% *mate45‐1* seedlings (Figure 7a,b, see Supporting Information Figure S7, arrowhead marks leaf primordia), and in ~40% of seedlings by 8 dag (compare Figure 7c,d). Different from WT, GUS staining was not maintained in the cotyledons of developing *mate45‐1* seedlings (see Supporting Information Figure S7). Together, these results showed that *mate45‐1* accumulated ABA in leaf primordium and gradually failed to maintain ABA levels in adjacent cotyledon tissues. To determine whether *mate45‐1* had reduced sensitivity to ABA in the cotyledons or other tissues we treat 4‐day‐old *mate45‐1 pRD29B::GUS* with 1 μM ABA for 24 hr. Cotyledons stained strongly but we observed weaker staining in the extravascular tissues of the hypocotyl and root, whereas WT seedlings showed uniform staining in these tissues (Figure 7e). Taken together with prior results, these suggest that *mate45‐1* exhibits altered seedling development and anthocyanin accumulation in AIC due to aberrant distribution and maintenance of ABA and its signaling.

To determine whether MATE45 could transport ABA directly, we incubated *E. coli acrB* cells expressing MATE45^long^, or the Venus fluorescent protein as a control, with 0.5 μM (+)‐ABA, and compared rates of ABA uptake into the cells by LC‐MS^n^. Three independent experiments demonstrated low, but significantly higher rates of ABA uptake into *E. coli* cells expressing MATE45^long^ compared to the control (*p *<* *0.05, two‐tailed students *t*‐test) (Figure 7f). Yet, the rates were roughly 1,000 times lower than those observed for ABCG25 (Kuromori et al., 2010). Based on these results, we conclude that MATE45 is unlikely to function as a bona fide ABA transporter.

### 4.7 Abscisic acid participates in the induction of anthocyanins in AIC

**Figure 8:**
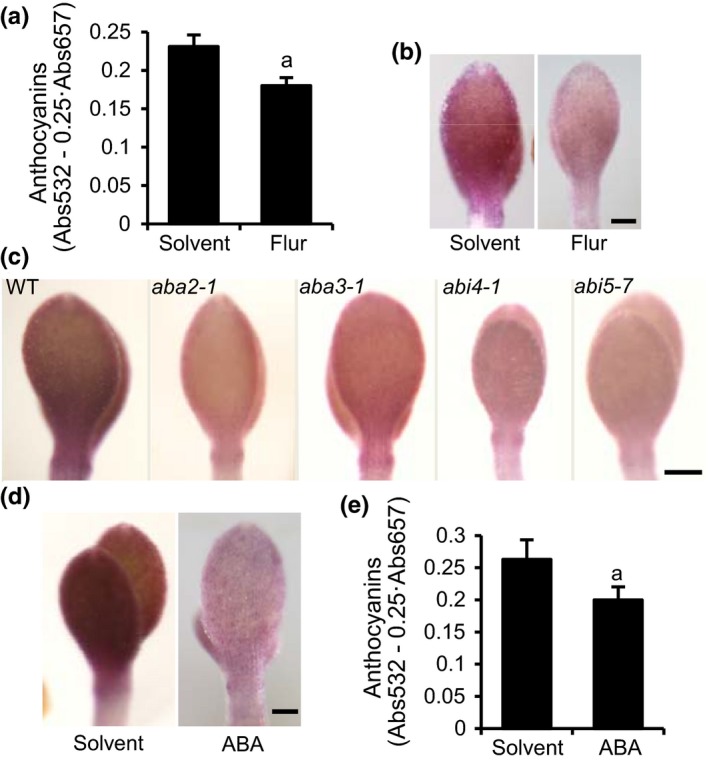
Abscisic acid (ABA) has a role in the induction of anthocyanins in response to AIC stress. (a) Total anthocyanin absorbance from the methanolic extracts of seedlings treated with ethanol (solvent) or 10 μM of the ABA biosynthesis inhibitor fluridone (Flur). Seedlings were grown for 3 days prior to the addition of chemical to the AIC medium for 24 hr. Error bars represent standard error of mean, *n* = 4 biological replicates. (b) Anthocyanin pigmentation phenotypes of seedlings from (a). Scale 125 μm. (c) Anthocyanin pigmentation phenotypes of mutants of ABA biosynthesis (*aba2‐1*,* aba3‐1*) and signaling (*abi4‐1*,* abi5‐7*) at four dag in AIC. Scale 125 μm. (d) Anthocyanin pigmentation phenotypes of wild‐type seedlings after treatment with ethanol (solvent) or 5 μM ABA for 24 hr in AIC. Scale 125 μm. (e) Total anthocyanin absorbance from the methanolic extracts of seedlings in (d). Error bars represent standard error of mean, *n* = 4 biological replicates

Since *mate45‐1* was identified in a screen for transporter mutants that had altered anthocyanin pigmentation, we sought to determine whether ABA was involved in the induction of anthocyanins in AIC. We treated WT seedlings with 10 μM Flur. After 24 hr, we observed a ~20% reduction in the levels of anthocyanins and a pale pink coloration in the epidermis (Figure 8a,b), like the pigmentation observed in the transporter mutants identified by our screen (Figure 1a). Further, the ABA biosynthesis deficient mutants *aba2‐1* and *aba3‐1* (Gonzalez‐Guzman et al., 2002; Xiong, Ishitani, Lee, & Zhu, 2001) and the *abi4‐1* and *abi5‐7* signaling mutants also showed pale anthocyanin pigmentation (Figure 8c). These results indicate that ABA plays a role in inducing anthocyanin pigmentation under AIC. However, treatment with 1–10 μM of ABA in AIC medium did not restore WT anthocyanin levels in *aba2‐1* or *aba3‐1*, or in any of the transporter mutants identified by our screen. ABA (at concentration as low as 1 μM) induced GUS staining in *pRD29B:GUS* (see Supporting Information Figure S8), confirming that the ABA treatments were effective. Interestingly, the cotyledons of WT seedlings treated with ABA for 24 hr showed pale pink anthocyanin pigmentation in the epidermal cells (Figure 8d) and reduced anthocyanin levels (Figure 8e). This implies that the induction of anthocyanin biosynthesis in epidermal cells was driven by distribution or transport patterns unique to endogenous ABA that are not mimicked by exogenous ABA treatment.

## 5 DISCUSSION

Our results identify *Arabidopsis* MATE45 as a meristem‐ and vascular‐localized metabolite transporter that has important functions in antagonizing ABA signaling in growing aerial meristems and in maintaining ABA signaling in adjacent non‐meristematic tissues. *MATE45* corresponds to *AtBIGE1a*, one of the two putative orthologs of *BIG EMBRYO1* (*BIGE1*), which was recently demonstrated to be involved in regulating the timing and rate of maize organ initiation (Suzuki et al., 2015). While the molecular function of BIGE1 is not known, it was proposed to participate in the transport of a metabolite, possibly part of the CYP78A pathway (Suzuki et al., 2015). Our findings establish that MATE45 produces at least three isoforms by alternative splicing, but that only one of them (MATE45^long^) has transporter activity. Thus, it possible that the three splice forms have different functions. Although *mate45* was identified because of a reduction in anthocyanin accumulation under nutritional stress, the perturbation in flavonoid accumulation is not responsible for the developmental phenotypes of *mate45*, which include delayed germination, reduced rosette and silique growth, and increased numbers of inflorescence branches and buds. By contrast, these developmental phenotypes were found to be ABA‐dependent since introducing the *aba2‐1* mutation, that confers reduced rates of ABA biosynthesis, fully rescued almost all phenotypes. Previously, the *aba2‐1* mutant revealed dual roles for ABA in plant development in the absence of severe stress; inhibiting germination modulated by sugars and osmotic signals, and promoting organ and body size and fertility (Cheng et al., 2002). In roots, ABA inhibits the growth of lateral meristems but stimulates or inhibits growth of the primary root meristem in a concentration‐dependent manner (Cheng et al., 2002). The concentration‐dependent role of ABA in promoting organ and body size remains poorly understood. Here, we demonstrated that *mate45‐1* and *aba2‐1* mutants have opposite phenotypes of leaf primordia growth in AIC and in dormancy under non‐stressed conditions. *mate45* dormancy was hypersensitive to ABA and primordia growth in AIC could be rescued by treatment with the ABA biosynthesis inhibitor norflurazon or by the *aba2‐1* mutation. Further, most developmental phenotypes of *mate45* in the absence of stress were fully rescued by *aba2‐1*. These results suggest that *MATE45* and *ABA2* pathways are mutually antagonistic and that WT phenotypes arise from a balance between the two. Localization of *MATE45* promoter activity to growing leaf and inflorescence meristems and to the plant vasculature indicates that those tissues are the sites of *MATE45* action. Since the vasculature is the predominant location of ABA biosynthesis (Endo et al., 2008), changes in anthocyanin pigmentation observed in non‐vascular cotyledon and hypocotyl tissues of seedlings in AIC were likely due to reduced ABA biosynthesis and export from the vasculature in *mate45* conferred by the reduced expressions of *NCED9*,* NCED3,* and *ABCG25*, respectively, which in turn caused less induction in anthocyanin biosynthesis and cumulatively less anthocyanins.

### 5.1 MATE45 function at the Golgi and TGN

The intertissue routes of ABA transport that are involved in stimulating stomatal closure during drought, and seed dormancy during imbibition, have been elucidated mainly by the tissue localization and phenotypes of plasma membrane‐localized proteins that mediate the cellular import or export of ABA. Promoter activities of *ABA‐IMPORTING TRANSPORTER* (*AIT*) *1*/*NPF4.6*, which also characterized as the low affinity nitrate transporter *NRT1.2* (Huang, Liu, Lo, & Tsay, 1999), were observed around the vascular tissues of inflorescence stems, leaves, and roots (Kanno et al., 2012). Compared to WT, the *ait1/nrt1.2* mutants were less sensitive to exogenously applied ABA during seed germination and/or post‐germination growth, whereas overexpression of *AIT1/NRT1.2* resulted in ABA hypersensitivity under the same conditions. Interestingly, the inflorescence stems of *ait1/nrt1.2* had excess water loss from open stomata (Kanno et al., 2012). The fact that the loss‐of‐function ABA import into the vasculature resulted in open stomata (Kanno et al., 2012) suggests that directional intertissue movement of ABA to the vasculature is a mechanism required to maintain WT levels of ABA that are needed for normal physiological responses. Since WT levels of guard cell response to dehydration requires both guard cell autonomous ABA synthesis and the export of vascular‐derived ABA (Bauer et al., 2013; Kuromori et al., 2010, 2014), this further supports the existence of intertissue feedback mechanisms that sustain and/or enhance ABA biosynthesis. *MATE45* promoter activity was localized to leaf primordia and vascular tissues in AIC and staining of the ABA reporter *pRD29B::GUS* in the *mate45* mutant revealed excess ABA signaling in the leaf primordium and reduced ABA levels in extravascular tissues. While it is conceivable that MATE45 functions to import cytoplasmic ABA into the TGN and Golgi for exocytosis from leaf primordium cells, thus redistributing it to adjacent tissues, the low rates of ABA transport observed for MATE45^long^ expressed in *E. coli* suggest that ABA is not the primary substrate. While we cannot rule‐out that MATE45 is post‐translationally modified or processed in the plant cell to enhance its affinity for ABA or for an ABA derivative, the studies in maize with BIGE1 (Suzuki et al., 2015) suggest the possibility that MATE45 is involved in a separate, antagonistic signaling pathway involving CYP78A and its derived metabolite. According to this hypothesis, MATE45 could function to efflux the CYP78A derived metabolite from TGN vesicles into the cytosolic space of leaf primordia or vascular cells to stimulate the antagonism of ABA signaling. Abrogation of this process in *mate45* could have caused excessive ABA signaling in leaf primordia or vascular cells. This in turn could have led to feedback suppression of local ABA biosynthesis and export from the vasculature and leaf primordia cells, causing reduced expressions of *NCED9* and *NCED3* and *ABCG25*.

### 5.2 Anthocyanins provide convenient readouts connecting abiotic stress and signal transduction

In *Arabidopsis*, anthocyanins are induced by a number of abiotic stress conditions, including the nutritional stress imposed by AIC (Kovinich et al., 2014, 2015; Pourcel et al., 2010; Poustka et al., 2007). For pigment coloration, anthocyanins need to be transported to the acidic vacuole, but no *Arabidopsis* tonoplast anthocyanin transporters have yet been identified. We reasoned that, based on what is known in maize and grapevine (Gomez et al., 2009; Goodman et al., 2004), a screen for MATE and ABC transporters under AIC could result in the identification of candidate anthocyanin transporters, as well as in transporters involved in the transport, distribution, and homeostasis of metabolites that participate in stress responses. The screen resulted in the identification of four homozygous mutants (*mate45‐1*,* abcg25‐5*,* abcg9‐1,* and *abcg5‐1)*. However, genotyping found that only ~50% of the T‐DNA population that was screened was homozygous. We are currently working toward obtaining more homozygous mutants from this population to screen for functions in mediating the anthocyanin response to abiotic stress.

## CONFLICT OF INTEREST

The authors declare no conflict of interest.

## AUTHOR CONTRIBUTIONS

N.K. and E.G. designed the research and wrote the paper with advice from the other authors; N.K., A.C., Y.W., P.D. and J.A. performed the research; N.K., A.C., Y.W., M.O., P.D. and E.G. analyzed the data..
