# Supplementary material for: Arabidopsis MATE45 antagonizes local abscisic acid signaling to mediate development and abiotic stress responses
Source: Plant Direct. 2018 Oct 12;2(10):e00087. doi: 10.1002/pld3.87 (PMC6508792; doi:10.1002/pld3.87)
Supplement: Supplementary file 1 [file PLD3-2-e00087-s001.pdf]

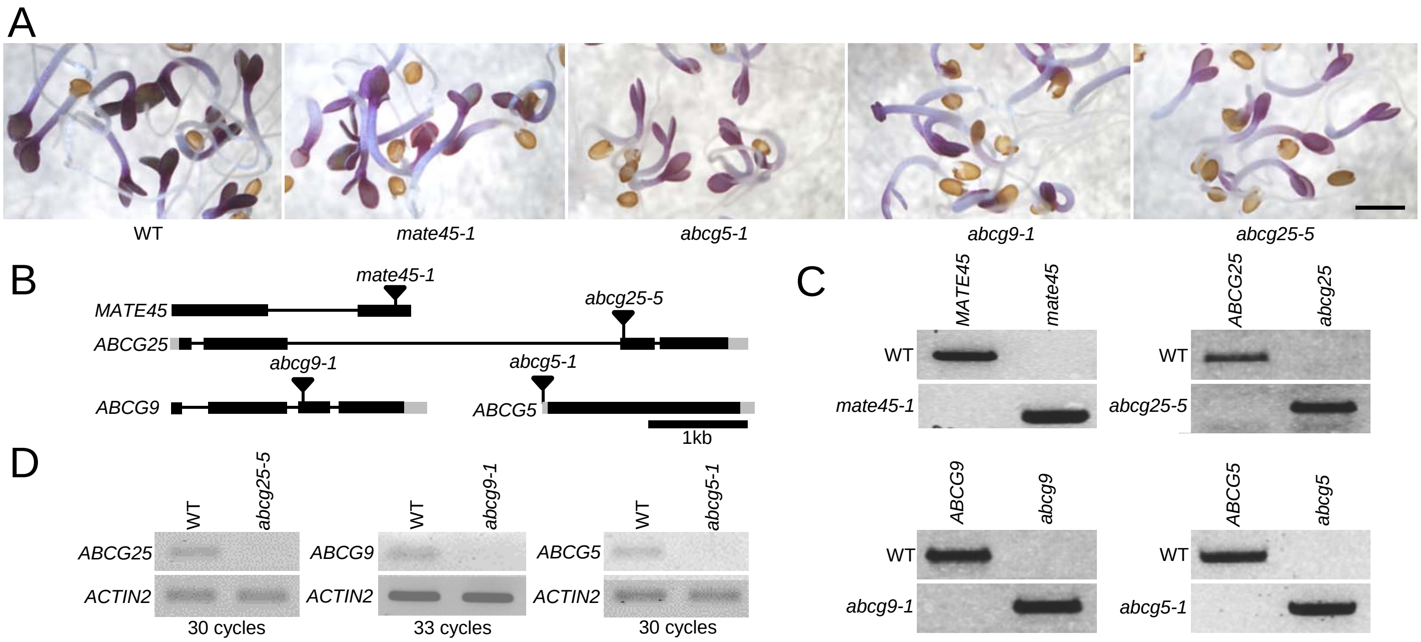

Supplemental Figure 1. Characterization of transporter gene mutants identified in a screen for altered anthocyanin pigmentation.

(A) Anthocyanin pigmentation phenotype of transporter gene mutants *mate45-1*, *abcg5*-1, *abcg9-1*, and *abcg25-5* identified by the AIC response screen had pale pink cotyledon color, whereas cotyledons of the Columbia wild-type (WT) had dark purple coloration. Seeds were germinated and grown in on a rotary shaker in 3% sucrose (w/v) in 24-well multititer plates under 24 h light for 5 days.

(B) Gene structures and insertional mutation sites of *mate45-1* (CS865060), *abcg5*-1 (SALK_074250C), *abcg9-1* (SALK_045397C), and *abcg25-5* (SALK_128873C). Black boxes represent exons, gray boxes untranslated regions, and black bars introns. T-DNA insertions are shown as triangles.

(C) Genotyping of the four mutants by PCR. Genomic DNA from wild-type (WT) or putative mutants (*mate45-1*, *abcg5*-1, *abcg9-1*, and *abcg25-5*) was isolated from pools of seedlings and genotyped by PCR using primers that target the wild-type (uppercase italic) or mutant (lowercase italic) alleles.

(D) Analysis of gene expressions in the mutants by RT-PCR. Total RNA was isolated from wild-type (WT) or putative mutants (*abcg25-5*, *abcg9-1*, and *abcg5-1*) grown in AIC for 4 days, and RT-PCR analysis was conducted using primers that anneal to the genes of interest (uppercase italic) or the endogenous reference *ACTIN2* (At3g18780) using the number of amplification cycles indicated. No PCR amplification was observed in control reactions where reverse transcriptase enzyme was omitted from the reaction mix (not shown). qRT-PCR analysis of *mate45-1* is shown in Figure 2B. Primer sequences are listed in Supplemental Table 2.
