# Supplementary material for: Arabidopsis MATE45 antagonizes local abscisic acid signaling to mediate development and abiotic stress responses
Source: Plant Direct. 2018 Oct 12;2(10):e00087. doi: 10.1002/pld3.87 (PMC6508792; doi:10.1002/pld3.87)
Supplement: Supplementary file 2 [file PLD3-2-e00087-s002.pdf]

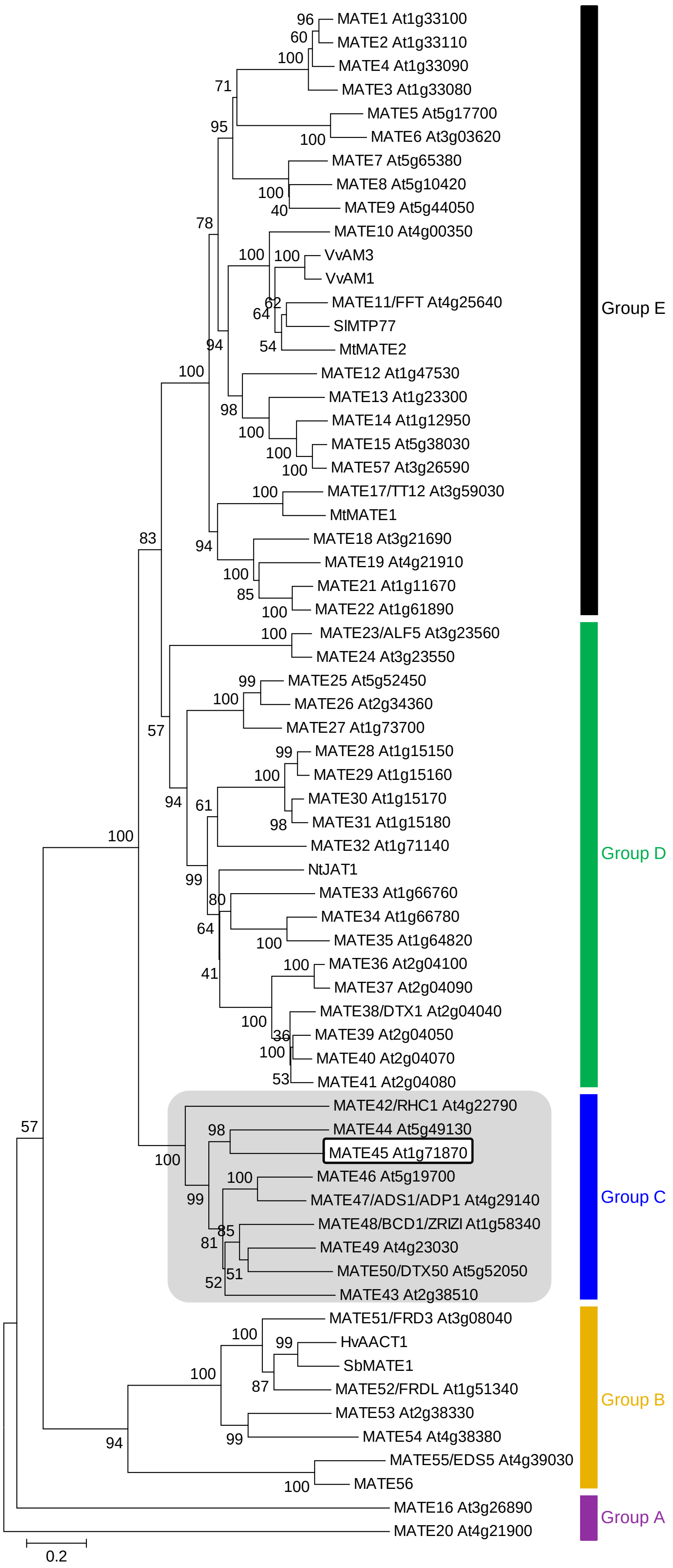

Supplemental Figure 2. Phylogeny of MATE transporters. Protein sequences of the 57 MATE transporters from Arabidopsis, including the characterized proteins DTX50, RHC1, EDS5, FRD3, FRDL, TT12, FFT, ALF5, DXT1, grapevine VvAM1 and VvAM3, Medicago truncatula MtMATE1 and MtMATE2, sorghum SbMATE1, tomato SlMTP77, barley HvAACT1, and tobacco NtJAT1 were aligned with ClustalW, and the nonrooted neighbor-joining tree was constructed using MEGA software (Version 5.05). Numbers at nodes indicate percent support from bootstrapping 10,000 times.
