# Supplementary material for: Arabidopsis MATE45 antagonizes local abscisic acid signaling to mediate development and abiotic stress responses
Source: Plant Direct. 2018 Oct 12;2(10):e00087. doi: 10.1002/pld3.87 (PMC6508792; doi:10.1002/pld3.87)
Supplement: Supplementary file 3 [file PLD3-2-e00087-s003.pdf]

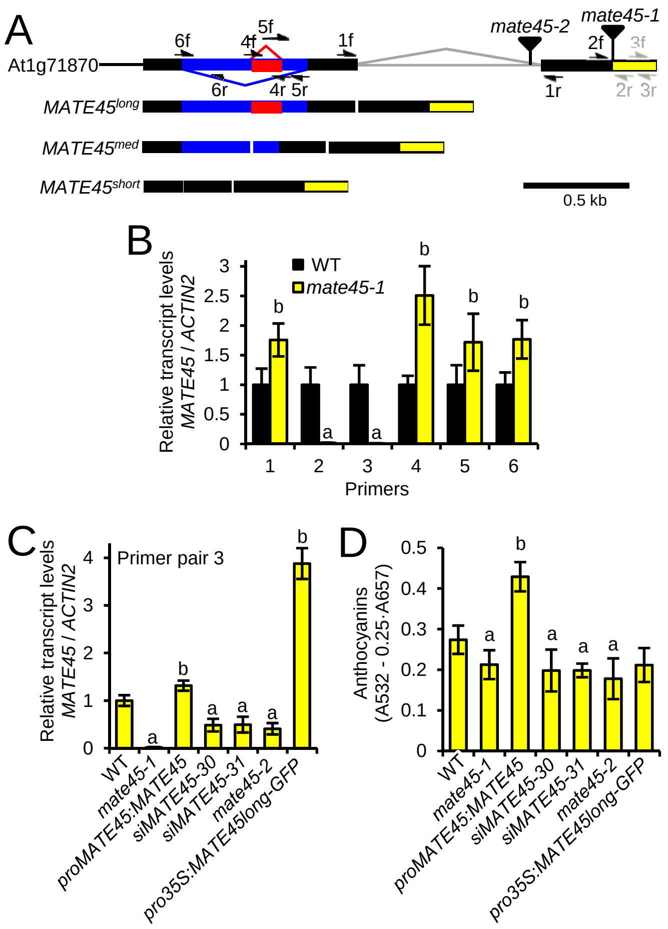

Supplemental Figure 3. Genetic characterization and anthocyanin levels of the *mate45* mutants and transgenic lines

(A) The *MATE45* gene structure, transcripts, and sites of insertional mutation for the three *mate45* alleles. Boxes represent coding regions, bars represent non-coding regions, triangles represent sites of *mate45-1* and *mate45-2* T-DNA insertions. The top schematic represents the *MATE45* gene, and three schematics below represent alternative mRNAs. Features shown are: promoter (black bar), constitutive intron (grey bar), splicing sites (angled bars), constitutive coding regions (black boxes), alternative coding regions (red and blue boxes), coding sequence knocked out by *mate45-1* T-DNA (yellow boxes), primers that bind the *MATE45* gene (black half arrows), primers that bind the *mate45-1* T-DNA sequence (grey half arrows).
(B) qRT-PCR measurement of *MATE45* expressions relative to *ACTIN2* in *mate45-1* using multiple primer pairs. WT values were set to 1. Primer binding sites are shown in Figure 2A. Total RNA was isolated from 4 day-old seedlings grown in AIC. Expressions were normalized to ACTIN2 (AT3G18780), relative expression levels are shown with WT values set to 1. Error bars represent the standard error of the mean (n = 4). [a]Less than control, [b]greater than control, $P < 0.05$; two-tailed Student's t test.
(C) qRT-PCR of *MATE45* mutants and transgenic lines. Primer pair 7-8 (see Figure 2A) was used to amplify cDNA from 4 day-old seedlings grown in AIC. Error bars represent the standard error of the mean (n = 6).
(D) Total anthocyanin levels of MATE45 mutants and transgenic lines. Spectrophotometry of methanolic extracts from 4 day-old seedlings grown in AIC. Error bars represent the standard error of the mean (n = 4). Seeds used in this assay were derived from plants grown under identical conditions and were harvested within on month of each other.
