# Supplementary material for: Arabidopsis MATE45 antagonizes local abscisic acid signaling to mediate development and abiotic stress responses
Source: Plant Direct. 2018 Oct 12;2(10):e00087. doi: 10.1002/pld3.87 (PMC6508792; doi:10.1002/pld3.87)
Supplement: Supplementary file 4 [file PLD3-2-e00087-s004.pdf]

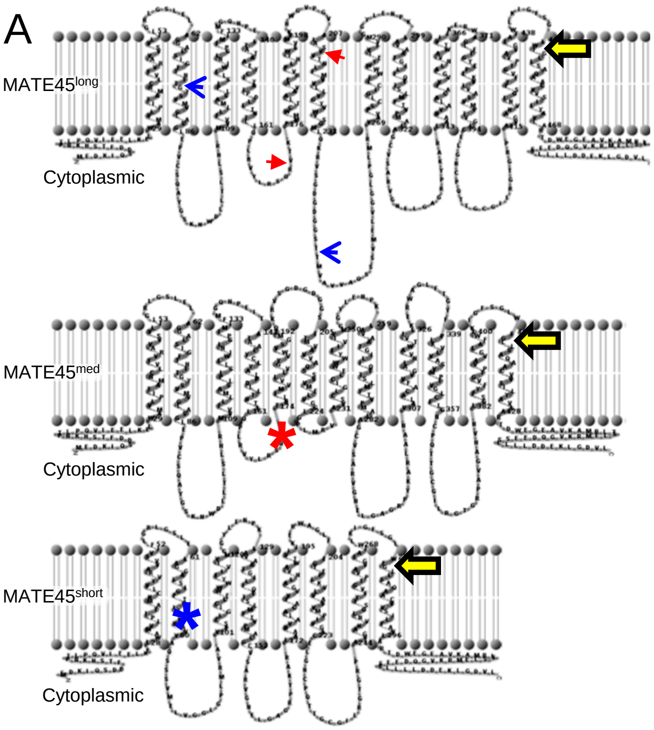

B

```
MATE45long_TMD12   445 GFSGLWFGLLSAQAACVVSIL 464
NorM-VC_TMD12      422 GAKGFWLGFIIGLSAAALMLG 441
                       * .*:*:*:: . :*..: :
```

Supplemental Figure 4. Secondary Structure of MATE45 Isoforms and Alignment of Deleted Residues of *mate45-1* with *NorM*.

(A) Predicted transmembrane domains of MATE45 isoforms. The peptide segments flanked by arrows in MATE45^{long} correspond to segments that are absent in MATE45^{med} (red) and MATE45^{short} (blue) due to alterative splicing. Stars mark the sites where peptide residues of MATE45long are absent. Everything C-terminal to the yellow arrow is putatively deleted in *mate45-1* due to the premature stop codon introduced by the T-DNA insertion. Transmembrane domain prediction by HMMTOP (http://www.enzim.hu/hmmtop/html/submit.html), drawn by TMRPres2D.

(B) Amino acid alignment of the deleted transmembrane domain of *mate45-1* with structurally characterized MATE NorM from *Vibrio Cholera*. Alignment with NorM-VC was done using ClustalW.
