# Supplementary material for: Arabidopsis MATE45 antagonizes local abscisic acid signaling to mediate development and abiotic stress responses
Source: Plant Direct. 2018 Oct 12;2(10):e00087. doi: 10.1002/pld3.87 (PMC6508792; doi:10.1002/pld3.87)
Supplement: Supplementary file 5 [file PLD3-2-e00087-s005.pdf]

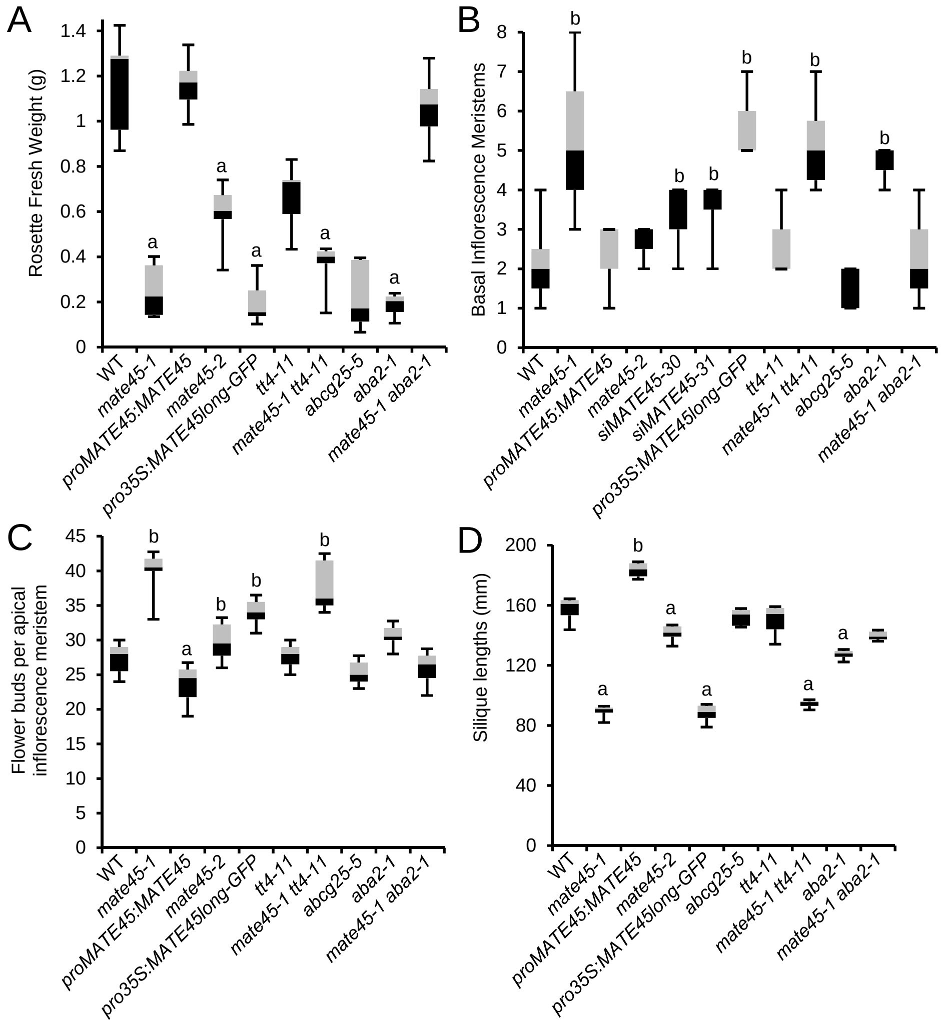

Supplemental Figure 5. Growth and Developmental Phenotypes of *MATE45* mutants.
(A) Rosette fresh weight was determined immediately after excision from the roots and inflorescence stems. n = 7-8 plants.
(B) Inflorescence stems originating from and growing more then 1 cm above the rosette base were counted. n = 7-8 plants.
(C) The flower buds per apical meristem were counted using a stereomicroscope. n = 7 plants.
(D) The length of siliques on the apical inflorescence meristem was measured. n = 7 plants, 10 siliques per plant.
Black blocks, second quartile; gray blocks, third quartile. Error bars represent the standard error of the mean. [a]Less than control, [b]greater than control, $P < 0.05$; two-tailed Student's *t* test. Plants were two months old, grown in long day conditions. See METHODS section for experimental details.
