# Supplementary material for: Arabidopsis MATE45 antagonizes local abscisic acid signaling to mediate development and abiotic stress responses
Source: Plant Direct. 2018 Oct 12;2(10):e00087. doi: 10.1002/pld3.87 (PMC6508792; doi:10.1002/pld3.87)
Supplement: Supplementary file 6 [file PLD3-2-e00087-s006.pdf]

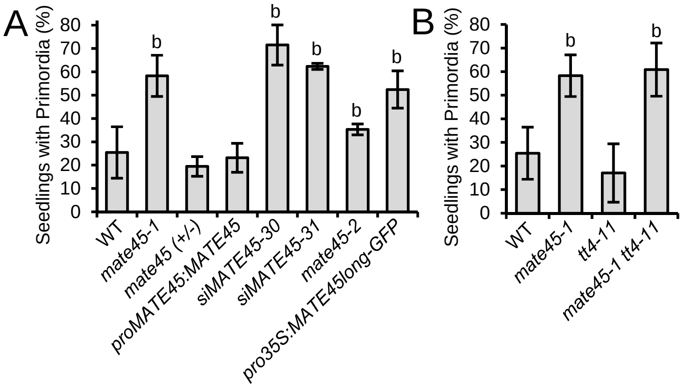


**Supplemental Figure 6.** *MATE45* Mutants had Accelerated True Leaf Primordia Growth during Growth in AIC Stress.

Percent of seedlings that had visible true leaf primordia at 12 dag in AIC . **(A)** MATE45 mutant and transgenic lines. **(B)** Flavonoidless mutants *tt4-11* and *mate45-1 tt4-11*. n = 4 biological replicates, ~120 seedlings per replicate. Error bars represent the standard error of the mean. [a]Less than control, [b]greater than control, $P < 0.05$; two-tailed Student's $t$ test.
