# Supplementary material for: Arabidopsis MATE45 antagonizes local abscisic acid signaling to mediate development and abiotic stress responses
Source: Plant Direct. 2018 Oct 12;2(10):e00087. doi: 10.1002/pld3.87 (PMC6508792; doi:10.1002/pld3.87)
Supplement: Supplementary file 7 [file PLD3-2-e00087-s007.pdf]

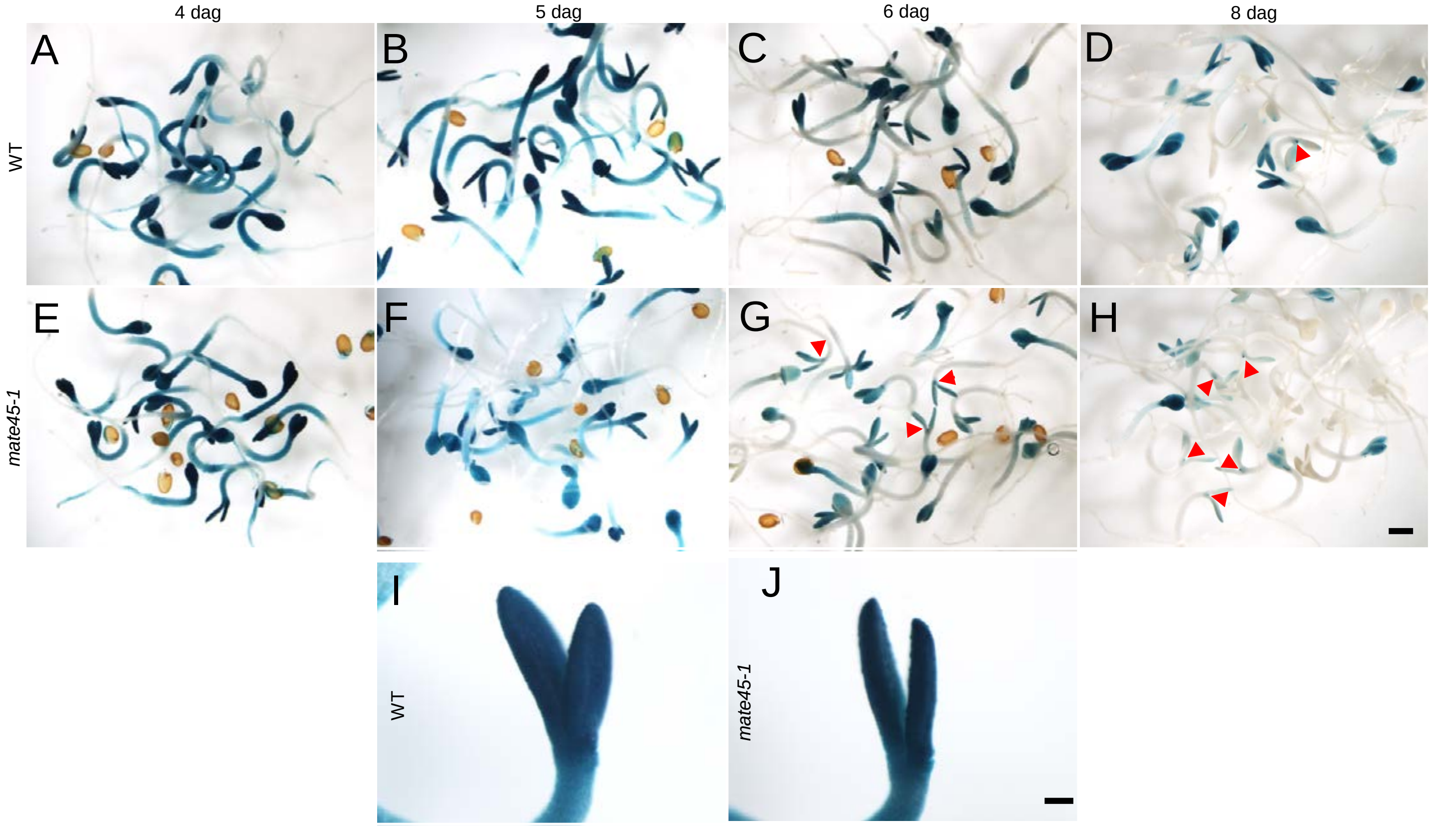

Supplemental Figure 7. Localization of ABA signaling in *mate45-1 pDR29B:GUS*.
(A – H) Effects of *mate45-1* on the distribution and relative intensities of the ABA marker *pDR29B:GUS* during seedling development in AIC. Wild-type seedlings (A – D) and *mate45-1* seedlings (E – H) expressing the ABA signaling marker *pDR29B:GUS* were imaged at 4 dag (A, E), 5 dag (B, F), 6 dag (C, G), and 8 dag (D, H) in AIC. Arrowheads mark regions of ectopic blue staining in leaf primordia. Note, ectopic staining was observed at much lower frequency in the WT consistent with a reduced frequency of primordia growth in WT (see Figure 7). Scale: 1 mm.
(I – J) Enlargement of seedlings at 4 dag showing roughly equal staining distribution. Scale: 125 µm.
