# Supplementary material for: Arabidopsis MATE45 antagonizes local abscisic acid signaling to mediate development and abiotic stress responses
Source: Plant Direct. 2018 Oct 12;2(10):e00087. doi: 10.1002/pld3.87 (PMC6508792; doi:10.1002/pld3.87)
Supplement: Supplementary file 8 [file PLD3-2-e00087-s008.pdf]

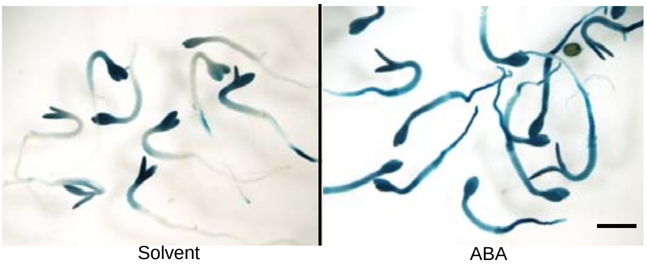

Supplemental Figure 8. Confirmation of ABA sensing.
Staining of ABA responsive marker line *pRD29B:GUS* (Christmann et al., 2005) after treatment with ethanol (solvent) or 1 µM ABA for 24 h in AIC. Scale 1 mm. The increased distribution of blue staining confirms that ABA treatment was inducing signaling.
