# Supplementary material for: Arabidopsis MATE45 antagonizes local abscisic acid signaling to mediate development and abiotic stress responses
Source: Plant Direct. 2018 Oct 12;2(10):e00087. doi: 10.1002/pld3.87 (PMC6508792; doi:10.1002/pld3.87)
Supplement: Supplementary file 13 [file PLD3-2-e00087-s013.pdf]

Responses are in blue font

EDITOR COMMENTS:

Thank you for submitting to Plant Direct. In light of the reviewers' comments transferred from our sister journal, minor editorial revisions are needed before the paper can be accepted for publication in Plant Direct.

No additional experiments are needed. Therefore you don't need to respond to the comments of reviewer 2 but you may want to consider to add in the discussion that the three spliced forms may have different functions. Please correct/clarify all points raised by reviewer 1. In addition,
1. In line 53, "and" should be "an".
2. In line 103, localized plasma membrane protein that "is" required

1 and 2 were corrected and the following sentence was added to the discussion where it was mentioned that only the MATE45long splice form had transporter activity; 'Thus, it possible that the three splice forms have different functions'.

> REVIEWER COMMENTS:
>
>
> Reviewer: 1
>
> The authors investigated an uncharacterized member of MATE transporter family. The genetic screening to observe anthocyanin pigmentation in AIC identified four transporter genes including MATE45. Anthocyanin pigmentation in AIC was related to ABA level and ABA signaling. MATE45 was expressing at meristem sites and vasculature, and localizing in subcellular TGN and Golgi. ABA biosynthesis and ABA signaling were disturbed in mate45 mutant, and the growth and development phenotypes were ABA-dependent, not due to anthocyanin level or other flavonoids. The authors showed the toxin transport activity and slightly ABA transport activity of MATE45, and also discussed the possibility of transporting other metabolite in CYP78A pathway or anthocyanin directly. Finally, a role of MATE45 was suggested in antagonizing meristematic ABA signal.
>
> In MATE45 functional analysis, the work is still incomplete to understand the substantial function of MATE45, for example, what is the substrate transported by MATE45. It is also not very clear to connect stress response, meristem growth and MATE45 functions on local ABA signaling followed by anthocyanin accumulation. However, several types of experiments were actually well performed to examine mutant phenotypes, double mutants appearances, ABA signaling induction, and so on. This work provides certain information and the manuscript is well written. The several following questions may help improving this manuscript.
>
> L149, L498 and Supplemental Table1
> It is difficult to understand which lines were actually screened. Why is there no MATE line in Supplemental Table1? Why were only four homozygous mutants in ABC family used, and three of the four lines identified as candidates? A reference to the supplemental table was added and the

supplemental table was corrected. The initial Supplemental file had an error (likely during conversion to PDF) and most of the table was missing.
>
> L174 and Figure1
> Are any anthocyanin types equally contributing the pigmentation? Or, A11 has a main effect? What is the difference between A11 and A11+? Please put a simple explanation. The following was added; 'Highly decorated cyanidin derivatives such as A11 and its isomer A11* contribute a deeper purple hue to tissues than less decorated cyanidin derivatives.'
>
> L462
> It will be better to eliminate "from guard cells". It is not yet proved that ABA synthesized at guard cells is moved to vasculature. Removed.
>
> L481-483
> Please check this sentence grammatically. Corrected.
>
> L812 and Table1
> What is the second column "MATE45 mate45-1"? Is it a heterozygote or a complemented line? This complemented line was described earlier in the text, however we did add a description in Table 1.
>
> Figure6
> It looks like much anthocyanin accumulation in mate45-1 and abcg25-5 in Figure6A. Is it compatible with the screening result? Yes, the mutants biosynthesize less anthocyanins but retain them longer. It is indicated in the figure caption that Fig. 6 was at 10 dag whereas the screen (Fig. 1) was at 4 dag.
>
> Figure7
> Should be one red box shown in Figure7E upper panel. Corrected.
>
> Supplemental Figure7
> Red arrow heads marks regions of ectopic blue staining in Supplemental Figure7G and H. It looks there are also at least two regions of ectopic blue staining in the center in Supplemental Figure7D. Please check it. We added a red arrow to Supplemental Figure 7D and the following note was added to the figure legend to remind the reader; 'Note, ectopic staining was observed at much lower frequency in the WT consistent with a reduced frequency of primordia growth in WT (see Figure 7).'
